# A qualitative evidence synthesis of participant, caregiver, and provider experiences of lung cancer exercise programs

**DOI:** 10.1007/s00520-025-09687-0

**Published:** 2025-07-07

**Authors:** Georgina A. Whish-Wilson, Lara Edbrooke, Catherine L. Granger, Emma Kinnersly, Alisha da Silva, Evelyn Sloan, Dominic Truong, Michelle Yi, Selina M. Parry

**Affiliations:** 1 Department of Physiotherapy, School of Health Sciences, The University of Melbourne, Alan Gilbert Building, Level 6, 161 Barry St, Carlton, VIC 3053 Australia; 2 Department of Health Services Research, Peter MacCallum Cancer Centre, 305 Grattan Street, Melbourne, VIC 3000 Australia; 3 Department of Oncology, Sir Peter MacCallum, The University of Melbourne, Parkville, VIC 3010 Australia; 4 Department of Physiotherapy, The Royal Melbourne Hospital, 300 Grattan Street, Parkville, VIC 3052 Australia

**Keywords:** Qualitative evidence synthesis, Exercise, Lung cancer, Prehabilitation, Rehabilitation, Thoracic surgery

## Abstract

**Purpose:**

This review sought to synthesise the existing qualitative literature to answer the question: “What are the experiences of people with lung cancer, caregivers, and/or providers regarding participation in or delivery of exercise programs?”.

**Methods:**

A qualitative evidence synthesis. Published literature from databases EMBASE (Ovid), MEDLINE (Ovid), PsycINFO (Ovid), CINAHL (EBSCO), Cochrane Library, Scopus, PEDro, and Web of Science and grey literature using thesis repositories and Google Scholar were searched on 18th February 2022 and updated on 8th October 2024. Included studies’ methodological limitations were evaluated using the Critical Appraisal Skills Programme (CASP) checklist by two independent researchers. First- and second-order qualitative data were extracted, cross-checked, and combined in thematic synthesis by one researcher, with another coding 10%, and they collaborated to determine the final themes, subthemes, and findings. Two independent researchers assessed the confidence in the findings using the GRADE-CERqual approach.

**Results:**

Twenty-four studies were included comprising 23 unique exercise programs (10 supervised centre-based and 13 unsupervised, home-based programs.). All studies included patients’/participants’ perspectives; six included clinicians, and two included caregivers. Forty-one findings were organised under four broad themes: 1) components of exercise program design, 2) providers of exercise programs, 3) the value of exercise programs, and 4) facilitating behaviour change. Overall, key stakeholders viewed exercise programs as effective and acceptable and valued individualised and tailored programs. Most findings were of moderate-to-high confidence.

**Conclusion:**

Based on the experiences of key stakeholders, 15 specific recommendations were generated that may improve the acceptability and effectiveness of lung cancer exercise programs.

**Supplementary Information:**

The online version contains supplementary material available at 10.1007/s00520-025-09687-0.

## Introduction

A strong quantitative evidence base supports the efficacy of exercise programs in improving outcomes for people with lung cancer [1]. Among those with operable lung cancer, preoperative exercise programs can reduce the incidence of postoperative pulmonary complications, reduce hospital length of stay, and improve exercise capacity and lung function [2]. Postoperative programs can improve exercise capacity, quadriceps muscle strength, health-related quality of life (HRQoL), and dyspnoea [3]. Lower certainty evidence supports the role of exercise among people with advanced disease, which may include improving exercise capacity, physical function, and HRQoL [1].

Implementation of exercise into routine lung cancer care remains poor worldwide [4–6]. Given these challenges, our focus must shift towards implementation strategies, including determining the most feasible, acceptable, effective, and cost-effective exercise delivery methods. Alongside the growing quantitative evidence base supporting the efficacy of these programs, there has been growth in the number of qualitative studies exploring patient perspectives and preferences for participating in such programs, further triangulating their utility and importance [7–9]. Qualitative evidence provides critical context regarding stakeholders’ experiences, priorities, and narratives, including factors that influence intervention acceptability, compliance, and uptake [10]. Similar to quantitative systematic reviews, qualitative evidence synthesis can increase the generalisability and transferability of individual qualitative studies, producing findings that clinicians can leverage to inform the development of patient-centred, context-sensitive interventions and evaluate how different intervention designs and components may influence outcomes [11, 12].

While several qualitative syntheses of exercise experiences exist in broader cancer populations [13–15], to our knowledge, no prior study has sought to synthesise the existing qualitative literature on exercise programs in a lung cancer population. Given people with lung cancer experience a unique and significant symptom burden compared to other cancers, and as such, specific barriers to exercise, it is important to explore their experiences in depth [16]. We also sought to include the experiences of the providers who deliver exercise programs and/or participants’ caregivers to provide further insight into moderators of program efficacy, adherence, and implementation.

The primary aim of this study was, therefore, to review and synthesise the existing qualitative literature to answer the research question “what are the experiences of people with lung cancer, caregivers and/or providers regarding participation in or delivery of exercise programs?” The secondary aim was to, where possible, investigate experiences based on program delivery (e.g., setting, supervision, exercise type or intensity) or participant characteristics (e.g., cancer stage or treatment intent).

## Methods

### Design

We conducted a qualitative evidence synthesis (QES) using Cochrane guidelines [17], reported in accordance with The Preferred Reporting Items for Systematic Reviews and Meta-Analyses (PRISMA) and the Enhancing Transparency in Reporting the Synthesis of Qualitative Research (ENTREQ) statements (Tables S1 and S2) [18, 19]. The protocol was prospectively registered with PROSPERO (CRD42022324779). Supplementary File S1 provides more detailed methods.

### Researcher reflexivity

Our team consisted of physiotherapists with experience working in lung cancer field and with positive views towards the role of exercise in lung cancer care. We minimised the influence of researchers’ positionally throughout data analysis wherever possible. Please see Supplementary File S1 for a more detailed reflexivity statement.

### Selection criteria

Eligible studies were published in English and used qualitative methods to collect and analyse data to investigate people with lung cancer’s experiences of participating in exercise programs (referred to as ‘participants’ henceforth) and/or their loved ones/caregivers or the providers involved in program delivery. Table 1 provides the detailed selection criteria.

**Table 1:** Selection criteria aligned to a modified Setting, Perspective, Intervention/Phenomena of Interest, Comparison and Evaluation (SPICE) Framework [17]

	Inclusion criteria	Exclusion criteria
**Setting**	• Any (e.g., inpatient, outpatient, home-based, telehealth, etc.) • Any geographical location	• Nil
**Perspective**	• Adults with confirmed or suspected lung cancer participating in an exercise program, and/or their caregivers or healthcare providers	• Mixed cohorts with < 50% lung cancer participants and qualitative findings not reported separately for lung cancer participants
**Intervention**	• Exercise programs that comprise more than 1 session • Any exercise type or intensity • Multimodal programs that include an exercise component	• Multimodal programs where < 50% participated in exercise and qualitative findings not reported separately for the exercise component • Intervention development studies where participants have not yet participated in a program
**Comparison**	• Nil	• Nil
**Evaluation**	• Participants’, caregivers’, and providers’ perceptions of an exercise program (e.g., experiences, preferences, barriers, facilitators, acceptability, feasibility, and effectiveness)	• Studies exclusively investigating aspects of intervention usability (e.g., appearance, readability, other digital usability metrics)
**Types of studies**	• Studies using qualitative methods for both data collection and analysis • Mixed methods studies where qualitative findings can be separately extracted from quantitative data • Available in English as full text	• Nil

### Search strategy and study selection

The search strategy was developed via an initial scoping review of the literature by researcher GAW-W to identify key terminology, followed by consultation with specialist librarians to finalise the search strategy. Search strategy development, including the use of qualitative search filters, was guided by the Cochrane Qualitative Methods Group [17]. The search strategy was optimised for each respective database before being applied to electronic databases EMBASE (Ovid), MEDLINE (Ovid), PsycINFO (Ovid), CINAHL (EBSCO), Cochrane Library, Scopus, PEDro, and Web of Science from inception (Tables S3-S10)[20]. Grey literature was searched for using Google Scholar and thesis repositories ProQuest Dissertations & Theses Global, EBSCO Open Dissertations, EThOS, and Open Access Theses and Dissertations (Table S11). The search was first run on 18th February 2022 and updated on 8th October 2024. Retrieved citations were imported into Covidence, where two independent researchers performed title, abstract, and full-text screening against the selection criteria (GAW-W and one of ADS, MY, or EK) with a third available if disagreements arose (SMP). De-duplication was conducted using software and by hand by GAW-W. Agreement (Cohen’s Kappa) between the researchers was calculated for the title/abstract and full-text screening stages.

### Assessment of methodological strengths and limitations

The methodological strengths and limitations (e.g., appropriateness and rigour of methodology, consideration of reflexivity and ethical principles, and clearness and validity of findings) of included studies were assessed by two independent researchers (GAW-W and ES) using the Critical Appraisal Skills Program (CASP) checklist, with disagreements resolved by a third (LE) [21].

### Data extraction

Important characteristic data (i.e., study characteristics [e.g., aims, design, methodology], participant characteristics [e.g., sex, age, lung cancer stage and treatment], and intervention characteristics guided by the TIDieR checklist [22] were extracted for each study using a bespoke data collection form [23]. First-order (participant quotes) and second-order data (researcher interpretation/analysis) were extracted from the included studies’ results sections before being imported into NVivo for synthesis [24, 25]. All extracted data were cross-checked by a second researcher (DT) [26].

### Data synthesis

Qualitative data were combined using thematic synthesis, as per the steps outlined by Thomas and Harden [27]. One researcher (GAW-W) completed inductive line-by-line coding of all included studies; a second researcher (EK) coded 10%. Each researcher independently created descriptive themes, before collaborating to iteratively determine the final analytical themes, subthemes, and findings. Subgroup syntheses were conducted to explore the factors influencing the perceptions of lung cancer exercise programs using in-built NVivo functions, whereby coding outputs were filtered based on participant characteristics (e.g., lung cancer stage/treatment intent) and/or intervention characteristics (e.g., program setting/timing, exercise type/intensity) and combined in thematic synthesis.

Given our research question pertained to participant experiences, no theoretical framework was specified a priori to guide the thematic synthesis. The researchers then reviewed the findings to identify important moderators of program effectiveness (e.g., successful behaviour change) and acceptability and create a list of program design and implementation recommendations. Key moderators were deductively identified from the findings and categorised using the Theoretical Domains Framework (TDF) and the Theoretical Framework of Acceptability (TFA) domains as guides before being formulated into actionable recommendations [28, 29].

### Assessment of confidence in the findings

The Grading of Recommendations Assessment, Development and Evaluation: Confidence in Evidence from Reviews of Qualitative Research (GRADE-CERQual) was used to assess the overall confidence in the review findings [30]. Each researcher independently rated their level of concern (from no/very minor to serious) for each component (methodological limitations, coherence, adequacy, and relevance) for each finding and then used this assessment to determine the overall level of confidence that the finding is a reasonable representation of the phenomenon of interest (from very low to high) [30]. Two researchers (GAW-W and EK) applied the GRADE-CERQual to each review finding independently, using published guidance, before collaborating to achieve consensus [30]. A third reviewer was available if disagreements arose, although was not required.

## Results

### Study identification

**Fig. 1:**
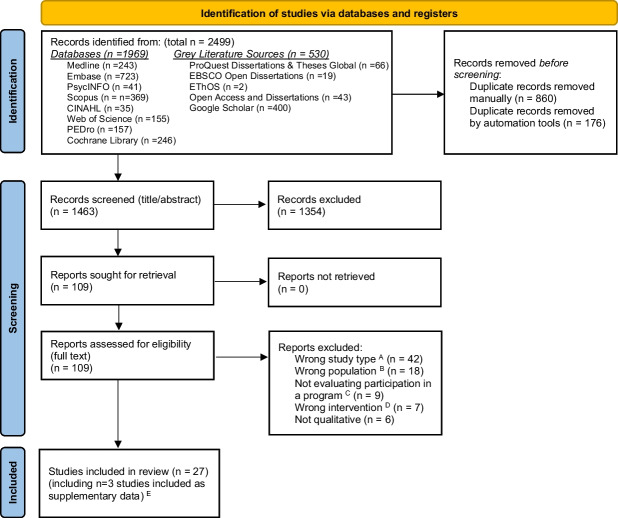
PRISMA flow diagram of study identification and selection. No additional records were identified through citation searching. The automation tool used for de-duplication was Covidence. ^A^ E.g., not available in English, conference poster or abstract only, book chapter, editorial providing no original data, clinical trial registry entry with no published data available. ^B^ E.g., < 50% of participants had lung cancer and qualitative findings from lung cancer participants were not reported separately. ^C^ E.g., studies that sought to develop a new program, investigate participants’ needs or perspectives relating to exercise/rehabilitation programs, or explore participants’ generic experience of exercise outside of a program. ^D^ E.g., program did not involve exercise or multi-modal programs where less than 50% of participants participated in the exercise component and findings from this cohort are not reported separately. ^E^ Additional published versions of studies already included that contained additional context and/or qualitative data (e.g., theses)

The search strategy identified 24 primary studies [7, 8, 31–52] (including 23 unique exercise programs) and three additional supplementary reports [53–55] (Fig. 1). Interrater agreement for title/abstract screening was 0.85, and for full-text screening was 0.82.

### Included studies

Forty-six percent of the studies were published since 2020. Sample sizes ranged from 5–37 (mean 14). Half of studies were conducted in Europe (50%) (Denmark ^(study #: 1, 15, 17, 18)^, England ^(2, 10, 14)^, Scotland ^(4)^, The Netherlands ^(5, 9, 24)^, Northern Ireland ^(19)^), with the rest from North America (38%) (Canada ^(3, 7)^, the United States ^(8, 11, 12, 16, 20, 22, 23)^), Oceania (8%) (Australia ^(6, 21)^) or Asia (4%) (China^(13)^). Based on the studies that reported participant demographics, patient age ranges ranged from 40 to 80s, with 60s being the average; and 53% of patients were female.

Just over half of studies included patients undergoing surgical management ± neo/adjuvant therapies (58%) ^(2, 4, 5, 8, 11, 12, 13, 14, 17, 18, 21, 22, 23, 24)^, whereas 21% focused on predominantly inoperable populations ^(1, 6, 7, 15, 19)^, 13% on mixed populations ^(3, 9, 20)^, and 8% did not report a primary population or treatment intent ^(10, 16)^.

Most studies (75%) collected qualitative data via semi-structured interviews ± focus groups or questionnaires/surveys ^(1, 2, 3, 4, 5, 6, 7, 8, 10, 13, 14, 15, 16, 17, 18, 19, 21, 22)^. Content analysis (29%) ^(6, 8, 11, 12, 14, 22, 23)^ and thematic analysis (29%) ^(1, 2, 5, 13, 16, 19, 21)^ were the most common qualitative data analysis methods. All studies included participants’ perspectives, 25% included providers ^(2, 3, 4, 15, 19, 24)^, and 8% caregivers ^(16, 20)^. Provider disciplines varied between studies, and included nurses (nurse navigators ^(15)^, clinical trials nurses ^(19)^, and unspecified/general ^(2, 4)^), physicians (respiratory ^(2)^, oncologist ^(19)^, and unspecified/general ^(2)^), physiotherapists ^(4, 15, 24)^, surgeons ^(2)^, advanced therapist practitioners ^(2)^, and ‘program facilitators’ ^(3)^. One study also included survivor advocates ^(3)^. Caregiver relationship to patients was reported in one study ^(20)^ and included friend (33%), spouse (56%), and other family member (22%).

**Fig. 2:**
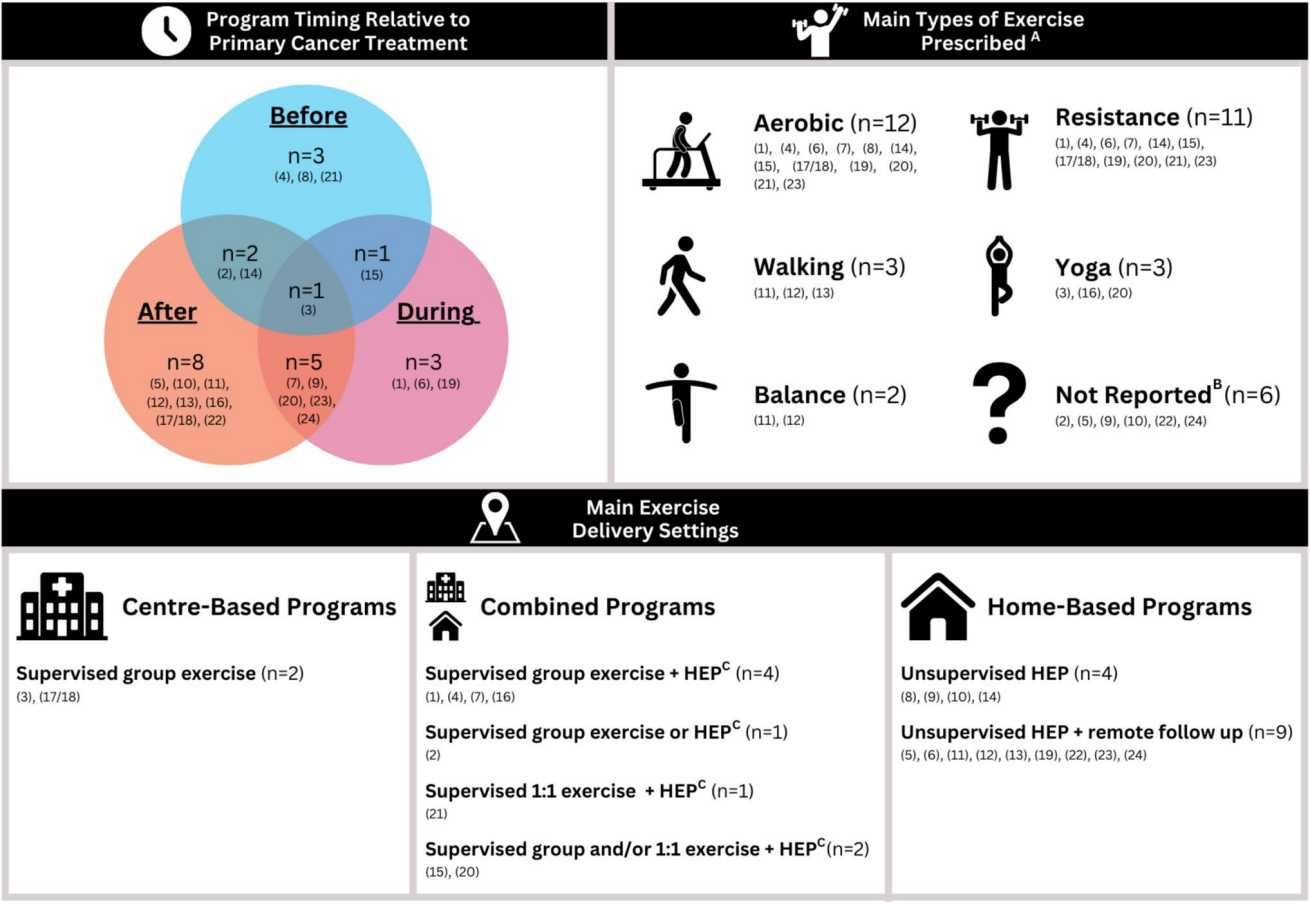
Summary of key exercise program characteristics of included studies (n = 24 studies, n = 23 unique programs). Abbreviations: HEP = home exercise program; (#) = study number. ^A^ Studies may have prescribed more than one main type of exercise. Studies that reported prescribing ‘aerobic’ exercise may have also included walking. ^B^ E.g., generic guidance to increase PA levels, ‘personalised exercise/rehabilitation recommendations,’ etc. ^C^ Unsupervised/independent HEP

Figure 2 provides an overview of exercise program characteristics. The most common exercise setting was home-based (unsupervised ± remote follow-up) (57%) ^(5, 6, 8, 9, 10, 11, 12, 13, 14, 19, 22, 23, 24)^, with 43% offering supervised, centre-based programs ^(1, 2, 3, 4, 7, 15, 16, 17/18, 20, 21)^. A combination of aerobic and resistance training was the most common exercise type prescribed (43%) ^(1, 4, 6, 7, 14, 15, 17/18, 19, 21, 23)^. Tables S12-15 provide more detailed characteristic information of included studies.

### Assessment of methodological strengths and limitations

The assessment of methodological strengths and limitations is provided in Table 2. The most common methodological limitations included reporting/discussion of researcher reflexivity and relationship with participants, recruitment (e.g., how a subgroup of program participants was chosen to participate in the qualitative evaluation), data collection reporting or rigour, and data analysis reporting. Strengths included clear statements of research aims and ethical considerations.

**Table 2:**
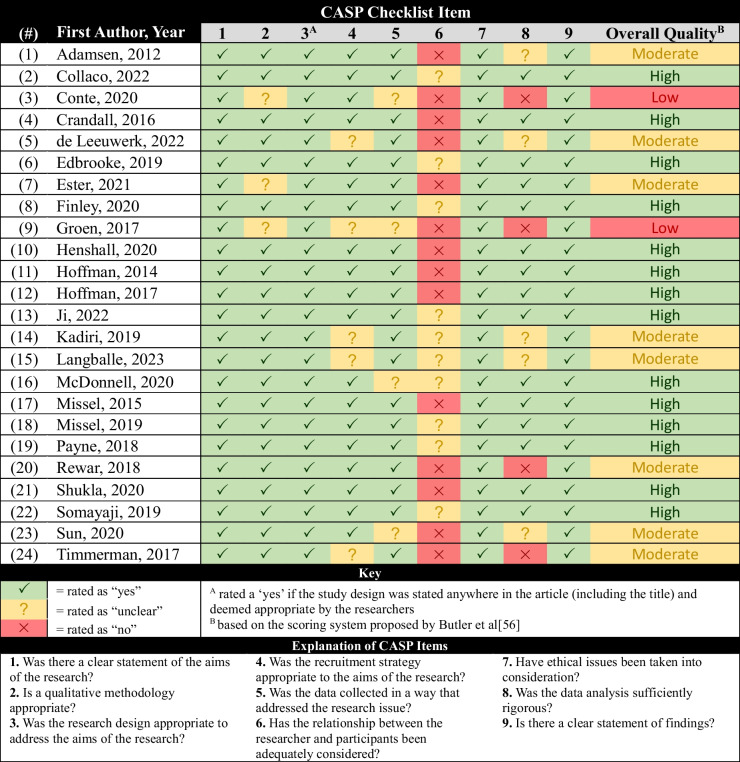
Methodological strengths and limitations of included studies using the CASP checklist [21]

### Thematic synthesis

Four overarching themes (1. Components of exercise program design, 2. Providers of exercise programs, 3. The value of exercise programs, and 4. Facilitating exercise behaviour change), 12 subthemes and 41 findings were developed from the thematic synthesis. These are represented in detail in Table 3. Table S16 provides supporting qualitative data.

**Table 3:** Summary of qualitative findings

#	Summarised review finding	GRADE-CERQual assessment of confidence	Explanation of GRADE-CERQual assessment	Supporting Studies
Theme 1: Components of exercise program design				
*Exercise prescription*				
1	Programs that prescribed exercise at an ‘achievable’ intensity and provided ‘controlled exposure’ to exercise supported participants in building exercise self-efficacy. Participants also appreciated provider-led exercise progression throughout the program	Moderate confidence	Due to minor concerns regarding methodological limitations and coherence, moderate concerns regarding adequacy, and no/very minor concerns regarding relevance	(4) (6) (7) (11) (16)
2	Participants valued individualisation and tailoring, including individualised exercise prescription, goal setting, and education. Individualisation was seen as an enabler of exercise participation, and some participants gleaned additional motivation to participate in exercise by feeling individually cared for	High confidence	Due to minor concerns regarding methodological limitations and no/very minor concerns regarding coherence, adequacy, and relevance	(2) (4) (6) (7) (10) (19) (20)
3	Walking was an accessible, simple and enjoyable form of exercise highly valued by participants	Moderate confidence	Due to minor concerns regarding methodological limitations, no/very minor concerns regarding coherence and relevance, and moderate concerns regarding adequacy	(4) (6) (13)
4	Preferences regarding exercise types were variable, highlighting the importance of autonomy and providing various exercise options to allow for individual preferences. Variety was seen as an enabler of sustained exercise enjoyment and behaviour change, and some participants viewed programs that did not offer this flexibility as regimented and boring	High confidence	Due to moderate concerns regarding methodological limitations, minor concerns regarding coherence, and no/very minor concerns regarding adequacy and relevance	(4) (7) (8) (12) (14) (19) (20) (22)
5	Some participants and providers of pre- and rehabilitation programs desired a longer program and/or more frequent sessions because they believed that longer programs were more beneficial	Moderate confidence	Due to moderate concerns regarding methodological limitations, coherence and adequacy, and no/very minor concerns regarding relevance	(1) (4) (16) (19) (20)
*Exercise setting*				
6	Participants in group programs saw them as valuable and an essential source of motivation, socialisation, and support. One study discussed group size and reported that participants tended to prefer smaller group sizes to foster familiarity, intimacy, and closeness and reduce feelings of intimidation and overwhelm. Participants valued face-to-face supervision and opportunities for one-on-one support from program providers to ensure they completed prescribed exercises correctly. Some participants of centre-based programs/desired an individualised home exercise program to support behaviour change and independent exercise outside supervised sessions	Moderate confidence	Due to serious concerns regarding methodological limitations, minor concerns regarding coherence, and no/very minor concerns regarding adequacy and relevance	(1) (3) (4) (7) (16) (18) (20)
7	Participants of home-based programs saw them as valuable. Many, especially participants with more advanced lung cancer, viewed home-based exercise as less burdensome than attending centre-based programs	Moderate confidence	Due to moderate concerns regarding methodological limitations, adequacy, and relevance, and minor concerns regarding coherence	(1) (6) (15)
8	Participants of digital programs had mixed experiences, with some reporting drawbacks such as inadequate tailoring, flexibility, feedback, and support	Low confidence	Due to serious concerns regarding methodological limitations, minor concerns regarding coherence and relevance, and moderate concerns regarding adequacy	(5) (9) (10) (24)
*Outcome measures*				
9	Participants viewed participating in outcome measurement assessments as a way to improve motivation, resilience and self-efficacy by allowing participants to exceed their own expectations, demonstrating what is possible, and providing an objective measurement of their capacity and progress	High confidence	Due to moderate concerns regarding methodological limitations, no/very minor concerns regarding coherence and relevance, and minor concerns regarding adequacy	(4) (7) (15) (19)
10	Some participants desired provider feedback regarding their performance in outcome measurements and progress throughout the program	Moderate confidence	Due to moderate concerns regarding methodological limitations, no/very minor concerns regarding coherence and relevance, and moderate concerns regarding adequacy	(6) (9) (20)
11	Participants'views regarding the acceptability and utility of specific outcome measures were varied. In one study, participants and providers viewed physical outcome measures such as the Six-Minute Walk Test and the 60-s Sit-to-Stand Test as useful, relevant tools to monitor fitness and progress. Providers in another study reported that the Ekblom-Bak test was too burdensome for participants to complete. Participants of two studies found Patient Reported Outcomes (PROs) overly burdensome, cumbersome, and/or difficult to complete, whereas participants of another found them acceptable	Low confidence	Due to serious concerns regarding methodological limitations, minor concerns regarding coherence, moderate concerns regarding adequacy, and no/very minor concerns regarding relevance	(9) (12) (15) (19)
*Program timing*				
12	Participants of some prehabilitation-only programs desired program continuation and support postoperatively to guide them to resume exercise and normal activities due to postoperative symptoms and physical inactivity	Moderate confidence	Due to no/very minor concerns regarding methodological limitations, coherence, and relevance, and moderate concerns regarding adequacy	(4) (21)
Theme 2: Providers of exercise programs				
*Expertise*				
13	Participants valued program providers’ expertise independent of their specialty/discipline, including expert education, guidance, monitoring, and exercise progression, regression, and modification. Support from a qualified expert improved confidence, safety, and trust	High confidence	Due to moderate concerns regarding methodological limitations, and no/very minor concerns regarding coherence, adequacy, and relevance	(4) (6) (7) (10) (12) (13) (15) (17) (19) (20) (21) (22)
*Role*				
14	Providers played a key role in supporting, encouraging, and educating participants to exercise and were seen as key instigators of health behaviour change by acting as important sources of extrinsic motivation and supporting participants to build exercise self-efficacy	High confidence	Due to moderate concerns regarding methodological limitations, and no/very minor concerns regarding coherence, adequacy, and relevance	(2) (6) (12) (13) (15) (17) (19) (21) (22)
15	Participants saw providers as critical sources of support throughout their cancer journey, transcending the role of an ‘exercise provider.’ Providers'roles included acting as conduits between participants and the healthcare system, explaining complex medical concepts in accessible ways, answering questions, and simply caring and listening. Many participants recalled generating close relationships with their providers, supporting exercise behaviour change	High confidence	Due to minor concerns regarding methodological limitations, and no/very minor concerns regarding coherence, adequacy, and relevance	(2) (6) (11) (12) (13) (15) (22)
Theme 3: The value of exercise programs				
*Taking an active role in one’s own healthcare*				
16	For many participants, a diagnosis of lung cancer was a ‘call to action’ to take control of their health through exercise. Participating in exercise programs facilitated many participants'generation of an internal locus of control and taking an active role in their healthcare. For some participants, participating helped them remain positive and hopeful, provided an opportunity to distance or distract themselves from a lung cancer diagnosis, and supported a shift in mindset towards wellness and recovery rather than sickness	High confidence	Due to moderate concerns regarding methodological limitations, and no/very minor concerns regarding coherence, adequacy, and relevance	(1) (4) (5) (6) (7) (11) (12) (16) (18) (19) (21) (22)
17	Program components such as exercise prescription/advice, activity monitoring tools, and symptom self-management education facilitated habit and skill-building, the development of exercise self-efficacy, and a ‘toolbox’ that supported participants to exercise independently. For many participants, this translated into plans to remain physically active after program discharge	Moderate confidence	Due to moderate concerns regarding methodological limitations, minor concerns regarding coherence and adequacy, and no/very minor concerns regarding relevance	(2) (4) (11) (12) (13) (16) (17) (19) (20)
*Social connectivity*				
18	Lung cancer group programs facilitated important opportunities for participants to forge friendships, a sense of belonging, and camaraderie through shared experiences of a life-altering diagnosis. Participants reported specific benefits of group-based training, such as improvements to social wellbeing, contextualising their experiences, learning from others’ experiences, building hope, and a sense of freedom to discuss and share grief. In some instances, participants became critical sources of social support for each other	Moderate confidence	Due to serious concerns regarding methodological limitations, minor concerns regarding adequacy, and no/very minor concerns regarding coherence and relevance	(1) (2) (3) (4) (7) (16) (17) (18) (20)
*Enjoyment*				
19	Many participants found enjoyment in exercise participation	Moderate confidence	Due to minor concerns regarding methodological limitations and coherence, moderate concerns regarding adequacy, and no/very minor concerns regarding relevance	(4) (6) (7) (13) (19)
*Benefits to physical and mental health*				
20	Most participants felt that participation directly improved their motivation to be physically active, and/or their physical activity levels	Moderate confidence	Due to serious concerns regarding methodological limitations, and no/very minor concerns regarding coherence, adequacy, and relevance	(1) (2) (4) (5) (6) (7) (12) (13) (14) (15) (17) (19) (20)
21	The benefits of participating in exercise programs were seen to ‘go beyond’ improvements to physical health, with many participants, providers and caregivers recalling the multifaceted healing capacity of exercise. Many participants voiced benefits to their physical and/or mental health, which were seen as interconnected. Successfully completing an exercise session often contributed to a sense of instantly feeling ‘better.’ Physical benefits included reduced symptoms such as breathlessness, pain and fatigue; improved symptom self-management, physical function, functional capacity, fitness, muscle strength and/or tone, and flexibility; and prevention of deterioration/functional maintenance. Mental benefits included improved self-esteem, self-efficacy, coping skills, relaxation, cognition, positivity, emotional regulation, and reduced stress and anxiety	High confidence	Due to moderate concerns regarding methodological limitations, and no/very minor concerns regarding coherence, adequacy, and relevance	(1) (2) (3) (4) (6) (7) (11) (12) (13) (14) (15) (16) (17) (19) (20) (22) (23)
22	Many participants generated increased exercise self-efficacy through repeated exercise practice and supported exercise skill-building	Moderate confidence	Due to moderate concerns regarding methodological limitations and coherence, minor concerns regarding adequacy, and no/very minor concerns regarding relevance	(2) (4) (6) (7) (13) (15) (16) (17) (20) (22)
23	All stakeholder groups reported that caregivers benefitted broadly from participating and/or supporting their loved ones, including increased motivation to be physically active themselves, improved physical and/or mental well-being, and an opportunity to support and/or re-build relationships with their loved one. They also reported improved knowledge, such as an increased understanding of their loved ones’ health and diagnosis, and of ways to support and care for their loved ones	Moderate confidence	Due to moderate concerns regarding methodological limitations, coherence and relevance, and minor concerns regarding adequacy	(4) (12) (16) (18) (19) (20) (22) (23)
24	Some participants of pre- and rehabilitation programs believed that exercise was a significant driver in their surgical readiness and improving and/or hastening their postoperative recovery	High confidence	Due to minor concerns regarding methodological limitations and adequacy, and no/very minor concerns regarding coherence and relevance	(2) (4) (6) (13) (14)
25	Most, but not all, participants felt that they benefited from participating in programs. Providers also believed that programs were beneficial. Some participants reported no perceived benefits, and others felt they would have been active despite their enrolment in a program and, therefore, that the program was unnecessary for them	Moderate confidence	Due to serious concerns regarding methodological limitations, and no/very minor concerns regarding coherence, adequacy, and relevance	(1) (2) (3) (4) (5) (6) (7) (12) (14) (15) (17) (19)
Theme 4: Facilitating exercise behaviour change				
*Key enablers of exercise*				
26	Generating exercise habits and finding ways to integrate exercise into the daily routine were key enablers of lasting behaviour change. Program structure and a requirement to attend scheduled sessions were key extrinsic motivating factors for participation for some participants, whereas for others, the flexibility home-based programs offered to choose when to exercise was an enabler	High confidence	Due to moderate concerns regarding methodological limitations, and no/very minor concerns regarding coherence, adequacy, and relevance	(1) (2) (4) (5) (6) (7) (11) (12) (13) (19) (20) (22)
27	Program providers were seen as key enablers of participation. Many participants felt accountable or committed to their providers, facilitating adherence. Participants of centre-based programs reported relying on external supervision to sustain motivation. Conversely, for participants of home-based programs, regular remote reviews with providers, such as phone calls, served a similar purpose. Across all exercise settings, participants valued receiving feedback on progress and activity levels from providers. Education and reassurance from expert providers regarding the importance and safety of exercise were critical enablers of exercise uptake and adherence	Moderate confidence	Due to serious concerns regarding methodological limitations, and no/very minor concerns regarding coherence, adequacy, and relevance	(1) (2) (4) (5) (6) (7) (8) (9) (12) (13) (15) (17) (19) (22) (24)
28	Clear activity goals (e.g., daily step targets) can drive behaviour change, and many participants saw activity monitoring tools such as pedometers as critical sources of extrinsic motivation. These tools provided a visual tool, enabled the self-monitoring of activity levels and recovery, and served as proof of ability. Achieving and/or exceeding exercise goals was an important driver of self-efficacy and a sense of achievement	Moderate confidence	Due to serious concerns regarding methodological limitations, minor concerns regarding coherence, and no/very minor concerns regarding adequacy and relevance	(4) (5) (6) (8) (9) (11) (12) (13) (14) (17) (19) (20) (22) (23)
29	Positive outcome expectancy was key to motivation and hope among many participants. Participants and providers tended to report a shared understanding of the importance and efficacy of exercise for patients with lung cancer and other medical conditions. Believing that the program would positively influence health, well-being or longevity, and/or prevent deterioration/decline, was a strong enabler of program sign-up and adherence, and experiencing perceived benefits of participation was additionally a strong enabler of adherence/sustained exercise uptake	Moderate confidence	Due to serious concerns regarding methodological limitations, minor concerns regarding coherence, and no/very minor concerns regarding adequacy and relevance	(1) (2) (3) (4) (6) (7) (8) (12) (13) (14) (15) (16) (17) (19) (21) (23) (24)
30	Many participants saw having pre-existing exercise habits before their lung cancer diagnosis as a key enabler to recommencing and sustaining activity, exercise self-efficacy, and increased exercise intensity, particularly when facing barriers	High confidence	Due to minor concerns regarding methodological limitations, coherence, and adequacy, and no/very minor concerns regarding relevance	(1) (4) (6) (13) (17) (19) (23)
31	Optimising symptom control and support to monitor and self-manage symptoms in collaboration with providers and other healthcare professionals enabled exercise uptake and adherence. Some participants displayed an inherent persistence and self-efficacy to exercise despite severe symptoms and side effects, while others generated this through the support of their providers	High confidence	Due to moderate concerns regarding methodological limitations, and no/very minor concerns regarding coherence, adequacy, and relevance	(1) (2) (4) (6) (7) (10) (12) (13) (14) (19) (22) (23)
32	Encouragement and support from loved ones increased motivation for some participants. Participating in exercise alongside loved ones also increased motivation and enjoyment	High confidence	Due to minor concerns regarding methodological limitations and adequacy, and no/very minor concerns regarding coherence and relevance	(4) (6) (13) (19) (21) (20) (22) (23)
*Key barriers to exercise*				
33	Symptoms such as fatigue, pain, and breathlessness were commonly experienced barriers to exercising. Adjuvant therapy side effects similarly often prevented participants from exercising, and participants recalled the ‘destructive’ nature of adjuvant therapies (especially chemotherapy) to both their bodies and their daily routines. Side effects associated with exercising such as exacerbating symptoms or inducing muscle pain/fatigue, and comorbidities, also influenced participation. The impacts of these barriers varied, sometimes forcing exercise regression or modification and/or completely inhibiting participation	Moderate confidence	Due to serious concerns regarding methodological limitations, and no/very minor concerns regarding coherence, adequacy, and relevance	(1) (6) (8) (11) (13) (14) (15) (17) (19) (20) (21) (23)
34	Time was a barrier to exercising. The unpredictability of cancer treatment pathways, particularly planned and unplanned medical appointments, influenced participants’ ability to participate in both home and centre-based programs. Additionally, exercise was often deprioritised in favour of other competing priorities, such as employment, caregiving, and socialising	High confidence	Due to moderate concerns regarding methodological limitations, no/very minor concerns regarding coherence and relevance, and minor concerns regarding adequacy	(4) (6) (7) (8) (17) (19) (20) (23)
35	Poor weather (e.g., rain, wind and extreme heat and/or cold) inhibited many participants'ability and/or willingness to participate in home-based exercise	High confidence	Due to moderate concerns regarding methodological limitations and adequacy, and no/very minor concerns regarding coherence and relevance	(6) (8) (13) (15) (23)
36	For participants of one study, social and cultural beliefs about emphasising rest in recovery were a barrier to postoperative exercise	Low confidence	Due to no/very minor concerns regarding methodological limitations and relevance, moderate concerns regarding coherence, and serious concerns regarding adequacy	(13)
37	Participants recalled difficulty generating the intrinsic motivation required to ‘get moving’, often citing their own ‘laziness’ or lack of discipline as a barrier. Low exercise self-efficacy similarly prevented some participants from exercising. Participants of centre-based programs especially often reported challenges initiating independent exercise outside of their supervised sessions due to lacking motivation and/or confidence	Moderate confidence	Due to moderate concerns regarding methodological limitations, no/very minor concerns regarding coherence and relevance, and minor concerns regarding adequacy	(1) (2) (4) (6) (7) (13) (15) (16) (17) (19) (20) (22) (23)
38	Pre-existing habits relating to exercise influenced participation. Participants who reported having a relatively sedentary lifestyle before commencing their program felt this was a barrier to exercise uptake. In contrast, some participants who viewed themselves as active felt that an exercise program would not necessarily benefit them	Moderate confidence	Due to minor concerns regarding methodological limitations and coherence, moderate concerns regarding adequacy, and no/very minor concerns regarding relevance	(1) (2) (4) (23)
39	Intervention acceptability was a barrier to exercise participation, including participants’ lack of enjoyment of exercise, preference to remain sedentary, and/or disinterest in the type of exercise prescribed. A minority of participants felt that they did not require such a program, and/or that it would not help them. Some participants and providers also questioned intervention appropriateness at times, for example, the appropriateness of prescribing high-intensity exercise to participants early after surgery or those with short life expectancies	High confidence	Due to moderate concerns regarding methodological limitations, no/very minor concerns regarding coherence and relevance, and minor concerns regarding adequacy	(1) (6) (8) (12) (15) (19) (20) (23)
40	Participants and providers encountered several logistical barriers, often specific to intervention design. Participants of centre-based programs were limited by travel and parking requirements, with some living considerable distances away. Prehabilitation programs were limited by the time between diagnosis and surgery	Moderate confidence	Due to moderate concerns regarding methodological limitations and adequacy, minor concerns regarding coherence, and no/very minor concerns regarding relevance	(2) (4) (6) (15) (20)
41	Participants of programs incorporating a technological element, such as online symptom monitoring or activity tracking technologies, encountered specific barriers, such as a lack of digital skills and/or self-efficacy, discomfort, and a general dislike of the prescribed technology	High confidence	Due to minor concerns regarding methodological limitations and adequacy, and no/very minor concerns regarding coherence and relevance	(6) (8) (12) (13) (15) (19) (22)

#### Components of exercise program design

The exercise programs included in this synthesis were heterogeneous. Participants commonly reported appreciation for programs that commenced exercise at an achievable intensity and were progressed by providers over time ^(contributing study #: 4,6,7,11,16)^. This controlled exposure was critical for participants in developing and sustaining exercise self-efficacy. Furthermore, participants emphasised the importance of individualisation and tailoring in exercise prescription, goal setting, and education, recognising that exercise should not be one-size-fits-all ^(2,4,6,7,10,19,20)^. Walking was often preferred because it was accessible, simple, and enjoyable ^(4,6,13)^. Incorporating participant preferences, autonomy, and variety into exercise prescription was seen as an enabler of sustained enjoyment and adherence ^(4,6,8,12,14,19,20,22)^.

Participants and providers of both supervised centre-based and unsupervised home-based programs tended to view them positively. Most centre-based programs involved group training, which was viewed as a critical source of motivation and social support by participants and providers ^(1,3,4,7,16,18,20)^. Opportunities for one-on-one interactions with exercise providers and access to individualised home exercise programs further supported participants’ ability to participate in exercise and translate structured exercise participation into more sustained behaviour change and habits ^(1,3,4,7,16,18,20)^. Participants of home-based programs felt that this mode of delivery reduced the participation burden (e.g. flexibility at home, reducing travel/appointment burden) ^(1,6,15)^. Some participants, and in one study providers, of unsupervised (i.e., asynchronous) home-based programs supported by digital applications (e.g., web or phone-based apps) reported limitations such as inadequate tailoring, flexibility, feedback, and support ^(5,9,10,24)^.

Assessments provided participants with insight into their capacity and progress and contributed to improvements in resilience, motivation, and self-efficacy ^(4,7,15,19)^. Participants were sometimes frustrated when they did not receive formal feedback regarding their performance in these assessments ^(6,9,20)^. There was inadequate data regarding which outcome measures participants viewed as most acceptable or meaningful ^(9,12,15,19)^.

There was a mixed response regarding program duration, with some happy with the length and others desiring longer programs and/or more frequent sessions ^(1,4,16,19,20)^. Some participants of prehabilitation-only programs felt that they required ongoing support after cancer treatment ^(4,21)^.

#### Providers of exercise programs

Participants felt providers played a key role in supporting their ability to exercise. They emphasised the importance of providers’ expert lung cancer and exercise prescription knowledge in facilitating the development of confidence, safety, and trust rather than their specific clinical background or discipline ^(4,6,7,10,12,13,15,17,19,20,21,22)^. This was particularly seen in participants’ gratefulness for providers’ ability to provide expert education, guidance, monitoring, and exercise progression/regression/modification. The provider role was seen as supporting and encouraging participants’ exercise uptake and adherence through providing extrinsic motivation and supporting the development of exercise self-efficacy ^(2,6,12,13,15,17,19,21,22)^; and as a source of general support and guidance (e.g., acting as a conduit between the participant and the medical system, answering questions, and acting as a supportive listening ear) ^(2,6,11,12,13,15,22)^.

#### The value of exercise programs

Overall, most participants, caregivers, and providers viewed exercise programs as beneficial. Specific benefits reported included: facilitating participants to generate an internal locus of control and adopt an active role in their own healthcare ^(1,4,5,6,7,11,12,16,18,19,21,22)^; the development of exercise skills and a ‘tool-box’ to support sustained exercise behaviours ^(2,4,11,12,13,16,17,19,20)^; enjoyment ^(4,6,7,13,19)^; improved motivation and/or physical activity levels ^(1,2,4,5,6,7,12,13,14,15,17,19,20)^; improved exercise self-efficacy ^(2,4,6,7,13,15,16,17,20,22)^; enhanced surgical readiness and/or postoperative recovery ^(2,4,6,13,14)^ and mental and physical health benefits such as improved symptoms, symptom self-management, and physical functioning, and reduced stress/anxiety ^(1,2,3,4,6,7,11,12,13,14,15,16,17,19,20,22,23)^. Group programs contributed to specific benefits such as improved social well-being, the development of camaraderie, and an improved ability to contextualise participants’ own experiences ^(1,2,3,4,7,16,17,18,20)^. Caregivers also experienced physical and mental health benefits and improved knowledge regarding their loved ones’ health and diagnosis ^(4,12,16,18,19,20,22,23)^.

#### Facilitating behaviour change

Generating exercise habits was seen as an important enabler of exercise behaviour change and adherence. For participants of centre-based programs, the requirement to attend and participate in structured exercise was a key source of extrinsic motivation. In contrast, participants of home-based programs saw flexibility and an unstructured approach as key enablers ^(1,2,4,5,6,7,11,12,13,19,20,22)^. Program providers were important sources of extrinsic motivation, facilitating exercise adherence through a perceived sense of accountability or commitment. Some participants of centre-based programs relied on this external supervision. In contrast, regular follow-up review appointments (e.g., via phone) supplemented this and provided necessary intrinsic motivation to participants of home-based programs ^(1,2,4,5,6,7,8,9,12,13,15,17,19,22,24)^. Other important enablers included clear activity goals and activity monitoring tools (e.g., pedometers) ^(4,5,6,8,9,11,12,13,14,17,19,20,22,23)^; belief that participation may improve health, well-being and/or longevity, or mitigate functional deterioration ^(1,2,3,4,6,7,8,12,13,14,15,16,17,19,21,23,24)^; having pre-existing exercise habits ^(1,4,5,13,17,19,23)^; optimising symptom management ^(1,2,4,6,7,10,12,13,14,19,22,23)^; and encouragement and support from loved ones ^(4,6,13,19,21,20,22,23)^.

Barriers to exercise participation included symptoms and neo/adjuvant therapy side-effects ^(1,6,8,11,13,14,15,17,19,20,21,23)^; time and other commitments, particularly planned and unplanned medical appointments ^(4,6,7,8,17,19,20,23)^; poor weather (e.g., rain, wind and extreme heat or cold) ^(6,8,13,15,23)^; social and cultural beliefs regarding the importance of rest ^(13)^; limited intrinsic motivation and/or exercise self-efficacy ^(1,2,4,6,7,13,15,16,17,19,20,22,23)^; a pre-existing sedentary lifestyle and/or already viewing oneself as ‘active enough’ ^(1,2,4,23)^; logistical barriers such as travel and parking for centre-based programs and/or inadequate time between diagnosis and surgery to facilitate prehabilitation ^(2,4,6,15,20)^; and for programs with a technological element, digital skills, digital self-efficacy and/or individual preferences e.g., dislike for the prescribed technology ^(6,8,12,13,15,19,22)^. Intervention acceptability was sometimes a barrier, e.g., dislike of exercise, a preference to remain sedentary, or a belief that the program is unlikely to benefit or is not required. Additionally, participants and providers of some programs questioned the appropriateness of providing exercise to patients soon after surgery or with shorter life expectancies ^(1,6,8,12,15,19,20,23)^.

### Subgroup thematic syntheses

#### Exercise setting

Subgroup syntheses of exercise settings demonstrated that participants typically held positive views of centre- and home-based programs. This was true across different cancer stages/treatment intents. While some individual preferences emerged, no specific findings suggested that either model was viewed as more acceptable, feasible, or effective from the perspectives of participants, caregivers, or providers. However, group programs appeared to confer a specific benefit to social wellbeing not reported in individual programs. Participants and providers of different program designs face specific barriers and enablers, e.g., centre-based programs were associated with increased travel/appointment burden, whereas participation in home-based programs was limited by poor weather.

#### Cancer stage/treatment intent

No specific findings were identified in subgroup synthesis that suggested stakeholder experiences or preferences varied based on cancer stage or treatment intent. Only four studies included wholly advanced/inoperable populations ^(1,6,7,19)^. All were delivered during treatment, with one continuing post-treatment ^(7)^. Two were group programs ^(1,7)^, and two were home-based with remote follow-up ^(6,19)^. Exercising during treatment and both delivery modes appeared acceptable across all stakeholder groups. The reduced participation burden associated with home-based programs may have been more meaningful for those with advanced lung cancer. The social benefits of group-based programs, including the freedom to discuss mortality and morbid thoughts, may also have been more meaningful for this group. There was inadequate data to perform subgroup syntheses of participants with advanced and/or inoperable lung cancer’s experiences of digital programs. In one study that included a predominantly inoperable population ^(15)^, most participants chose an unsupervised home program rather than a centre-based program. In the same study, providers questioned the appropriateness of prescribing vigorous-intensity exercise to patients with a short prognosis. Barriers and enablers to exercise did not appear to differ across different cancer stages/treatment intents.

#### Exercise type/intensity

Due to the heterogeneity of included programs, limited reporting, and the relatively high satisfaction/acceptability reported by most studies, subgroup syntheses of exercise types and intensity were limited. While walking appeared to be a highly valued and accessible form of exercise, we did not identify any specific exercise type(s) that were more acceptable, feasible, or enjoyable than others. Our findings suggest that participants desire variety, choice and autonomy over the type of exercise they participate in. We did not identify any specific experiences unique to exercising at different intensities. However, our findings suggested that participants may find programs that commence at an ‘achievable’ exercise intensity more acceptable and sustainable.

### Summary of recommendations

Based on the deductive analysis of the qualitative findings, 15 key recommendations were developed for program design and implementation (Table 4).

**Table 4:** Recommendations for the development and implementation of patient-centred exercise programs for lung cancer based on the qualitative evidence

Recommendations	Supporting finding(s)
*Recommendations for Enhancing Program Acceptability*	
Program Design	
1. Programs should be individualised, tailored, and flexible regarding: a. Exercise prescription b. Goal setting c. Education d. Design (e.g., length, setting, level of supervision, delivery mode)	2–5, 26
2. Programs incorporating technology (e.g., web or mobile app-delivery programs, programs prescribing wearable technology) should: a. Investigate ways to ensure content is adequately tailored and flexible b. Consider incorporating opportunities (regular or ad-hoc) for participants to interact with providers in real-time c. Formally assess participants’ preferences, skills and self-efficacy regarding using digital tools and investigate ways to embed support to improve participants’ digital skills and self-efficacy	2, 8, 41
Exercise Prescription	
3. Programs should aim to provide controlled exposure to exercise by commencing at an achievable intensity and supporting participants to progress their exercises at appropriate intervals	1
4. Programs should explore ways to incorporate evidence-based exercise guidelines while: a. Allowing participants agency to select the types of exercise that they prefer b. Ensuring variety within the prescribed exercises	2–4
5. Programs should consider opportunities where possible to formally engage caregivers in exercise programs	21, 23
Outcome Assessment	
6. Chosen outcome measures should be relevant, meaningful and acceptable for participants, and participants should be provided feedback regarding their performance in outcome measures and progress throughout the program	9–11
*Recommendations for Enhancing Behaviour Change*	
Assessing and Overcoming Barriers	
7. Programs should seek to formally assess and understand participants’ individual exercise enablers and barriers to provide targeted solutions/education. This review lists common enablers and barriers that could serve as a starting point for such assessments (e.g., symptoms and side-effects, outcome expectancy, pre-existing exercise habits, motivation, social supports, etc.)	29–40
8. Programs should incorporate education and support regarding symptom management and self-management, and how to exercise safely despite symptoms and side-effects	27, 31, 33
9. Programs should incorporate education specifically targeted towards caregivers, focusing on increasing their understanding of their loved one’s health/diagnosis and capability to support/care for their loved one	23
Facilitating Sustained Habit Building	
10. Programs should prioritise strategies that facilitate participants to build exercise habits to facilitate sustained behaviour change. Providing clear activity goals and utilising activity monitoring tools appear to support participants in incorporating physical activity into their daily lives, self-monitor activity levels, and improving exercise self-efficacy	26, 28
11. Programs should provide participants with regular feedback on their progress and physical activity levels to facilitate sustained motivation/adherence and exercise self-efficacy	27
Providers	
12. Providers should have training and expertise in lung cancer and exercise prescription (e.g., exercise monitoring, progression, regression, and modification)	13–15, 27
13. Programs should aim for continuity of providers to support the development of a strong therapeutic relationship, confidence, safety, trust, and a sense of commitment/accountability	13–15
Setting-Specific Recommendations	
14. Centre-based programs should be supplemented with individualised home exercise programs, and group programs should consider ways to incorporate opportunities for one-on-one support	6
15. Home-based programs should incorporate regular reviews with providers (e.g., in person or via telehealth) to facilitate sustained motivation, ongoing symptom-management support, and exercise progression/regression/modification	7, 27

### Confidence in the findings

Of the 41 findings, 17 were rated high confidence, 21 moderate confidence, and 3 low confidence using the GRADE-CERqual approach (Table 3). Further explanation regarding each GRADE-CERqual assessment is provided in Table S17.

## Discussion

Participants, caregivers, and clinicians typically report positive experiences regarding participation in/delivery of lung cancer exercise programs, including high acceptability and perceived efficacy. These views were held regardless of patient-level factors (e.g., cancer stage, cancer treatment) and program-level factors (e.g., program timing or delivery method).

Several previous qualitative syntheses have examined the experiences of mixed cancer groups participating in exercise, including patients with advanced cancer [15], patients undergoing chemotherapy [13], and patients participating specifically in supervised moderate-to-vigorous intensity exercise [14]. Across these three prior studies, only two primary papers included in synthesis were conducted in a majority lung cancer population. In contrast, our study narrows the focus specifically to patients with lung cancer, while broadening the range of interventions and perspectives synthesised.

While some of our findings echo prior syntheses in highlighting benefits beyond physical health, such as enhanced autonomy, hope, optimism, and specific mental health benefits (e.g., reduced anxiety/stress, improved mood), our findings also reveal unique implications for the lung cancer population. Participants described exercise as a means of reclaiming agency in the face of a potentially poor prognosis, and recontextualising their identity beyond illness. Group-based programs also emerged as particularly valuable in improving social and mental wellbeing and reducing isolation, all particularly relevant and important findings in the context of lung cancer given the documented high stigma and self-blame, symptom burden, distress, and low survival rates in comparison to other cancers. As outlined by Andersen et al. group-based exercise provided a welcome reprieve from dwelling on diagnosis and/or prognosis, and important opportunities to debrief and share morbid thoughts [13]. In the context of lung cancer, particularly advanced/inoperable disease, this finding is likely of specific importance. In the absence of high-certainty quantitative evidence supporting the routine use of exercise in this subpopulation of patients with lung cancer, our qualitative findings highlight the important physical and psychological benefits of exercise experienced by patients with inoperable lung cancer and their caregivers. While limited prior research has explored the specific, quantitative benefits of group-based exercise compared to individual exercise in this population, improved social support does appear to improve the quality of life of people with lung cancer [56]. Our qualitative findings further support the need for models of lung cancer care that enhance social support and reduce isolation.

### Implications for clinical practice

Based on the experiences of key stakeholders, we have generated 15 specific recommendations that may improve the feasibility, acceptability, and effectiveness of lung cancer exercise programs. We recommend that, alongside the existing quantitative evidence base and clinical practice guidelines, clinicians consider our recommendations to guide the delivery of evidence-based, responsive, and holistic lung cancer exercise care.

Given the heterogeneity of included programs, we cannot draw conclusions on the optimal program design, and future quantitative and qualitative research should compare the feasibility, acceptability, and effectiveness of centre versus home-based exercise for people with lung cancer. Importantly, our findings suggest that both modes of exercise delivery appear acceptable and beneficial to participants across different contexts, each with specific benefits and drawbacks that should be considered in clinical decision making. Based on these findings, health services in rural/remote areas, or those with limited resources, may consider prioritising the development and implementation of remotely delivered options, whereas metropolitan centres may be able to adapt and/or expand existing programs and infrastructure to meet the needs of this population.

Despite centre-based programs currently being the most common model of exercise offered in clinical practice [4], most exercise programs included in our synthesis were conducted in the home-based environment. None of the included studies included synchronous/supervised telehealth exercise, a model investigated more extensively in the chronic respiratory disease/pulmonary rehabilitation population [57]. Given emerging evidence that supports the effectiveness of remotely delivered, home-based programs [58, 59], and the widespread poor implementation of centre-based programs [4], these programs may represent a more implementable model of care. Digitally delivered exercise interventions (e.g., telehealth, apps, and wearables) are also an emerging field, with evidence supporting their potential efficacy [60, 61]. Our findings highlight the potential role of these tools in enhancing exercise program accessibility, particularly in rural/remote regions, as well as important considerations such as digital literacy, preferences, and self-efficacy. Before implementing these tools, clinicians should formally assess these aspects and embed formal strategies such as upskilling and technical support within programs. Additionally, avenues for integrating peer support within remotely delivered programs should be explored, such as supervised telehealth programs, access to video or telephone-conferencing support groups, or web/app-based moderated message boards.

Based on our findings, regardless of delivery setting, clinicians should prioritise flexibility and individualisation by offering adaptable, hybrid models of exercise care that allow participants increased choice and agency and an opportunity to embed exercise around an unpredictable disease and treatment trajectory. Future research should also explore the feasibility and effectiveness of these models. Outside of flexibility in delivery mode, lung cancer exercise programs should embed responsive approaches to exercise prescription, such as mechanisms to regularly review and adapt exercise type and intensity alongside patient feedback, goals, and often unpredictable clinical fluctuations. Qualitative findings also demonstrate the importance of, and benefits to, caregiver involvement. As such, clinicians should consider avenues for incorporating caregivers into exercise programs, such as formal approaches (e.g., dyadic exercise), informal approaches (e.g., general encouragement to participate in home-based exercise alongside their loved one) and delivering individualised caregiver-specific education.

### Limitations

The review is limited by the methodological limitations of included studies and the brevity of some studies’ findings, including a lack of supporting quotations. The latter was mitigated by including 24 total studies in the synthesis, most of which contained rich supporting data. Some studies did not report important intervention and/or participant characteristics, which limited our ability to undertake subgroup analyses. By the nature of participant inclusion in the primary studies, we are likely to have a biased sample of participants who favour exercise. The views of those who declined to participate in exercise programs should be explored in future research. Given that most of the studies included were from Europe and North America, this limits the generalisability of our findings beyond these populations. Future research should examine the experiences of culturally and racially marginalised populations.

## Conclusion

This QES demonstrated that people with lung cancer, caregivers, and clinicians view exercise programs as acceptable and effective. The perceived benefits of programs were seen to extend beyond improvements to physical health. While overall preferences were variable, participants appear to value interventions that emphasise exercise individualisation and tailoring. We have moderate-to-high confidence in most of our findings, which can be used to inform the development and implementation of patient-centred exercise programs, and therefore improve outcomes, for people with lung cancer.

## Supplementary Information

Below is the link to the electronic supplementary material.

Supplementary file1 (DOCX 161 KB)

## Data Availability

No datasets were generated or analysed during the current study.
