# Supplementary material for: A qualitative evidence synthesis of participant, caregiver, and provider experiences of lung cancer exercise programs
Source: Support Care Cancer. 2025 Jul 7;33(7):664. doi: 10.1007/s00520-025-09687-0 (PMC12234642; doi:10.1007/s00520-025-09687-0)
Supplement: Supplementary file 1 — Supplementary file1 (DOCX 161 KB) [file 520_2025_9687_MOESM1_ESM.docx]

**Supplementary File S1: Methods Supplement**

*Researcher Reflexivity*

The authors of this review acknowledge that it is likely our backgrounds (e.g., subject expertise and research and clinical experiences) have influenced our approach to this review, including in determining our research question, and throughout protocol development, data collection, and analysis. Our team consists of physiotherapists with varying degrees of experience in the clinical management and research of patients with lung cancer (<1 year to >10 years). At the commencement of this review, all authors held positive views towards the role of exercise in lung cancer care. Wherever possible, we have aimed to minimise the influence of researchers’ positionality throughout data analysis.

Authors GAW-W and SMP undertook the Cochrane Australia ‘Qualitative Evidence Synthesis (QES) and Grading of Recommendations Assessment, Development and Evaluation: Confidence in Evidence from Reviews of Qualitative Research (GRADE-CERQual)’ online course in 2021 before commencing this review. GAW-W and EK conducted the thematic synthesis and GRADE-CERQual assessment. GAW-W (she/her, senior physiotherapist, PhD candidate), the lead author, is a clinician-researcher with 6 years of clinical experience and 5 years of experience in qualitative research methods. Throughout the conduct of this review, she worked clinically as a physiotherapist within inpatient and outpatient oncology/haematology and inpatient respiratory and cardiothoracic surgery units. EK (she/her) is a physiotherapist and research assistant with 5 years of clinical experience and 6 years of experience in qualitative research methods. Throughout the conduct of this review, EK worked clinically as a physiotherapist within the community health setting treating mostly musculoskeletal/orthopaedic/persistent pain conditions across paediatric and adult populations.

*Research Question and Aims*

The initial protocol for this review served to demonstrate its boundaries while acknowledging the iterative nature of searching and synthesising qualitative research. Through identifying available evidence and potential lines of inquiry, the authors continued to refine and refocus the protocol, research question and study criteria through iterative searching techniques, as recommended by the Cochrane Qualitative and Implementation Methods Group[1]. Throughout this iterative process, the protocol was updated regularly, with any adaptations and deviations transparently recorded and reported in the final manuscript[2].The research question and study protocol were finalised and prospectively registered with the International Prospective Register of Systematic Reviews (PROSPERO) before data extraction was commenced.

We hypothesised that available evidence might provide insight into opinions, experiences, personal preferences, barriers, and facilitators to exercise as well as overall intervention feasibility, appropriateness, meaningfulness, and effectiveness (as adapted from the Feasibility, Appropriateness, Meaningfulness and Effectiveness [FAME] framework)[3].

*Selection Criteria*

Study eligibility criteria were defined using the Setting, Perspective, Intervention/Phenomenon of Interest, Comparison, Evaluation (SPICE) Framework for research question design (Table S1)[4]. Studies that included intervention development and evaluation phases were eligible for inclusion, and data were only extracted from the latter section of the paper.

*Search Strategy and Study Selection*

Both published and non-published (i.e., grey literature) studies were searched for. The databases and repositories listed in the primary manuscript were searched from inception. The search was first conducted on 18^th^ February 2022, and re-run on the 8^th^ of October 2024 for all databases and repositories, except for EThOS (E-theses online service), as it has been unavailable since 2023 due to a cyber-attack. No limits were applied to the searches for date or language. Filters were used to limit results to qualitative papers. The InterTASC Information Specialists’ Sub-Group (ISSG) Search Filter resource was used to determine which filters to apply to each search to target qualitative literature[5]. Database specific search strategies are provided in Tables S2-S10. The Google Scholar search strategy was optimised for retrieving grey literature, and the first 200 results were retrieved[6, 7]. Cited references were searched for each included study by hand-searching each reference list.

The search strategy was supplemented by the CLUSTER approach (Citations, Lead authors, Unpublished materials, Scholar searches, Theories, Early examples, Related projects) to identify “study clusters” – ‘a group of related papers that explore and explain various features of a single project and thus supply necessary detail relating to theory and/or context’ [8]. No additional papers were identified for inclusion into the evidence synthesis through CLUSTER searching, however, we identified key groups of papers that supported our understanding of the different interventions included in the review (e.g., published protocols, papers reporting quantitative outcomes). We utilised all components of the CLUSTER approach except for reaching out to lead authors to locate unpublished materials (step 7).

The search was not peer-reviewed. De-duplication was undertaken by Covidence software and by hand by one author (GAW-W) (Figure 1, main manuscript).

*Data Extraction*

Before the commencement of searching, it was recognised that qualitative studies face reporting limitations due to journal word limits. Therefore, to ensure we had a thorough understanding of the characteristics of each included study, additional papers identified through the CLUSTER method were used to supplement data extraction. Where necessary data were not available in the primary paper, it was extracted from the relevant study registrations, protocols, theses, and/or other published papers where possible. We also extracted any additional unpublished qualitative data to be included in the thematic synthesis from these records (e.g., published theses that included more data than the final, published journal article). We did not contact any authors to obtain missing data.

*Data Synthesis*

Thematic synthesis was conducted using the steps proposed by Thomas and Harden:

1. Coding text: inductive line-by-line coding of qualitative data
2. Developing descriptive themes: individual codes analysed for their meanings, with related codes clustered into descriptive categories
3. Generating analytical themes: similar categories are merged and analysed in the context of the research question to ‘go beyond’ the original findings of each individual paper, generating new concept sand understandings of the observed phenomena.

It was planned a priori that, where possible, sub-group analyses would be conducted to categorise patient experiences based on intervention specifications, including disease state, mode of exercise delivery (e.g., education only, home-based, hospital/clinic setting, supervised vs. unsupervised); intervention intensity; and intervention timing (e.g., preoperative, postoperative programs). Alternative lines of enquiry were also planned pending the availability of relevant primary research, including the experiences and opinions of healthcare professionals and/or caregivers. Therefore, findings from the completed review may include combinations of these aspects, and the research question was iteratively adapted throughout the search process to reflect the decided line(s) of inquiry.

Finalising the findings was an iterative process involving two reviewers. While assessing our confidence in the findings (see below), we reworded six of them to enhance their coherence.

*Assessment of Confidence in the Findings*

We used the GRADE-CERQual approach to rate the confidence in our findings, using published guidance[9-14]. Two independent reviewers rated the level of concern (either no/very minor, minor, moderate or serious) within each criterion for each finding:

- **Methodological limitations:** The extent to which there are concerns about the design or conduct of the primary studies that contributed evidence to an individual review finding
- **Coherence:** An assessment of how clear and cogent the fit is between the data from the primary studies and a review finding that synthesises that data. By ‘cogent’, we mean well supported or compelling
- **Adequacy of data:** An overall determination of the degree of richness and quantity of data supporting a review finding
- **Relevance:** The extent to which the body of evidence from the primary studies supporting a review finding is applicable to the context (perspective or population, phenomenon of interest, setting) specified in the review question

These assessments were then combined to determine the overall confidence in the finding:

- **High confidence:** It is highly likely that the review finding is a reasonable representation of the phenomenon of interest
- **Moderate confidence:** It is likely that the review finding is a reasonable representation of the phenomenon of interest
- **Low confidence:** It is possible that the review finding is a reasonable representation of the phenomenon of interest
- **Very low confidence:** It is not clear whether the review finding is a reasonable representation of the phenomenon of interest

The GRADE-CERQual approach was conducted using the Interactive Summary of Qualitative Findings (iSoQ) tool[15]. The reporting of our GRADE-CERQual process and findings was informed by published guidance[16].

Table S1: Compliance with the PRISMA Statement [17]

| **Section and topic** | **Item #** | **Checklist item** | **Location reported** |
| --- | --- | --- | --- |
| **TITLE** | | | |
| Title | 1 | Identify the report as a systematic review. | Main manuscript, p.1 |
| **ABSTRACT** | | | |
| Abstract | 2 | See the PRISMA 2020 for Abstracts checklist. | Main manuscript, p.1 |
| **INTRODUCTION** | | | |
| Rationale | 3 | Describe the rationale for the review in the context of existing knowledge. | Main manuscript, p.2 |
| Objectives | 4 | Provide an explicit statement of the objective(s) or question(s) the review addresses. | Main manuscript, p.2 |
| **METHODS** | | | |
| Eligibility Criteria | 5 | Specify the inclusion and exclusion criteria for the review and how studies were grouped for the syntheses. | Main manuscript, Table 1 |
| Information sources | 6 | Specify all databases, registers, websites, organisations, reference lists and other sources searched or consulted to identify studies. Specify the date when each source was last searched or consulted. | Main manuscript, p. 5 |
| Search strategy | 7 | Present the full search strategies for all databases, registers and websites, including any filters and limits used. | Supplementary file, Tables S3-S11 |
| Selection process | 8 | Specify the methods used to decide whether a study met the inclusion criteria of the review, including how many reviewers screened each record and each report retrieved, whether they worked independently, and if applicable, details of automation tools used in the process. | Main manuscript, p. 5 and Supplementary File pp.1-2 |
| Data collection process | 9 | Specify the methods used to collect data from reports, including how many reviewers collected data from each report, whether they worked independently, any processes for obtaining or confirming data from study investigators, and if applicable, details of automation tools used in the process. | Main manuscript, p. 5 and Supplementary File p. 2 |
| Data items | 10a | List and define all outcomes for which data were sought. Specify whether all results that were compatible with each outcome domain in each study were sought (e.g. for all measures, time points, analyses), and if not, the methods used to decide which results to collect. | Main Manuscript p. 5 and Supplementary File pp.1-2 |
|  | 10b | List and define all other variables for which data were sought (e.g. participant and intervention characteristics, funding sources). Describe any assumptions made about any missing or unclear information. | Main Manuscript p. 5 and Supplementary File pp.1-2 |
| Study risk of bias assessment | 11 | Specify the methods used to assess risk of bias in the included studies, including details of the tool(s) used, how many reviewers assessed each study and whether they worked independently, and if applicable, details of automation tools used in the process. | Main manuscript, p. 5 |
| Effect measures | 12 | Specify for each outcome the effect measure(s) (e.g. risk ratio, mean difference) used in the synthesis or presentation of results. | N/A |
| Synthesis methods | 13a | Describe the processes used to decide which studies were eligible for each synthesis (e.g. tabulating the study intervention characteristics and comparing against the planned groups for each synthesis (item #5)). | Main Manuscript p. 5 and Supplementary File pp.1-2 |
|  | 13b | Describe any methods required to prepare the data for presentation or synthesis, such as handling of missing summary statistics, or data conversions. | Supplementary File p.2 |
|  | 13c | Describe any methods used to tabulate or visually display results of individual studies and syntheses. | N/A |
|  | 13d | Describe any methods used to synthesize results and provide a rationale for the choice(s). If meta-analysis was performed, describe the model(s), method(s) to identify the presence and extent of statistical heterogeneity, and software package(s) used. | Main Manuscript p. 5 and Supplementary File pp.1-2 |
|  | 13e | Describe any methods used to explore possible causes of heterogeneity among study results (e.g. subgroup analysis, meta-regression). | Main Manuscript p. 5 |
|  | 13f | Describe any sensitivity analyses conducted to assess robustness of the synthesized results. | N/A |
| Reporting bias assessment | 14 | Describe any methods used to assess risk of bias due to missing results in a synthesis (arising from reporting biases). | N/A |
| Certainty assessment | 15 | Describe any methods used to assess certainty (or confidence) in the body of evidence for an outcome. | Main Manuscript p. 6 and Supplementary File p.3 |
| **RESULTS** | | | |
| Study selection | 16a | Describe the results of the search and selection process, from the number of records identified in the search to the number of studies included in the review, ideally using a flow diagram. | Main Manuscript Figure 1 |
|  | 16b | Cite studies that might appear to meet the inclusion criteria, but which were excluded, and explain why they were excluded. | Main Manuscript Figure 1 |
| Study characteristics | 17 | Cite each included study and present its characteristics. | Main Manuscript Table 2, Supplementary Tables S12-15 |
| Risk of bias in studies | 18 | Present assessments of risk of bias for each included study. | Main Manuscript Table 2 |
| Results of individual studies | 19 | For all outcomes, present, for each study: (a) summary statistics for each group (where appropriate) and (b) an effect estimate and its precision (e.g. confidence/credible interval), ideally using structured tables or plots. | N/A |
| Results of syntheses | 20a | For each synthesis, briefly summarise the characteristics and risk of bias among contributing studies. | Main manuscript, Table 3 |
|  | 20b | Present results of all statistical syntheses conducted. If meta-analysis was done, present for each the summary estimate and its precision (e.g. confidence/credible interval) and measures of statistical heterogeneity. If comparing groups, describe the direction of the effect. | N/A |
|  | 20c | Present results of all investigations of possible causes of heterogeneity among study results. | N/A |
|  | 20d | Present results of all sensitivity analyses conducted to assess the robustness of the synthesized results. | N/A |
| Reporting biases | 21 | Present assessments of risk of bias due to missing results (arising from reporting biases) for each synthesis assessed. | N/A |
| Certainty of evidence | 22 | Present assessments of certainty (or confidence) in the body of evidence for each outcome assessed. | Main manuscript, Table 3 and Supplementary Table S17 |
| **DISCUSSION** | | | |
| Discussion | 23a | Provide a general interpretation of the results in the context of other evidence. | Main manuscript, pp. 21-23 |
|  | 23b | Discuss any limitations of the evidence included in the review. | Main manuscript, pp. 22-23 |
|  | 23c | Discuss any limitations of the review processes used. | Main manuscript, pp. 22-23 |
|  | 23d | Discuss implications of the results for practice, policy, and future research. | Main manuscript, pp. 21-23 |
| **OTHER INFORMATION** | | | |
| Registration and protocol | 24a | Provide registration information for the review, including register name and registration number, or state that the review was not registered. | Main manuscript, p. 3 |
|  | 24b | Indicate where the review protocol can be accessed, or state that a protocol was not prepared. | A protocol was not prepared. |
|  | 24c | Describe and explain any amendments to information provided at registration or in the protocol. | N/A |
| Support | 25 | Describe sources of financial or non-financial support for the review, and the role of the funders or sponsors in the review. | Statement of financial support provided. |
| Competing interests | 26 | Declare any competing interests of review authors. | No competing interests to declare. |
| Availability of data, code and other materials | 27 | Report which of the following are publicly available and where they can be found: template data collection forms; data extracted from included studies; data used for all analyses; analytic code; any other materials used in the review. | N/A |

Table S2: **Compliance with the ENTREQ statement [18]**

| **#** | **Item** | **Description** | **Location Reported** |
| --- | --- | --- | --- |
| 1 | Aim | State the research question the synthesis addresses. | Main manuscript p.1 |
| 2 | Synthesis methodology | Identify the synthesis methodology or theoretical framework which underpins the synthesis and describe the rationale for choice of methodology. | Main manuscript p.5 and Supplementary File p.2 |
| 3 | Approach to searching | Indicate whether the search was pre-planned or iterative. | Main manuscript p.5 and Supplementary File pp.1-2 |
| 4 | Inclusion criteria | Specify the inclusion/exclusion criteria*.* | Table 1 |
| 5 | Data sources | Describe the information sources used and when the searches conducted; provide the rationale for using the data sources. | Main manuscript, p. 5, Supplementary File p.1-2 |
| 6 | Electronic search strategy | Describe the literature search. | Supplementary file, Tables S3-S11 |
| 7 | Study screening methods | Describe the process of study screening and sifting*.* | Main manuscript, p. 5, Supplementary File p.1-2 |
| 8 | Study characteristics | Present the characteristics of the included studies. | Supplementary Tables S12-15 |
| 9 | Study selection results | Identify the number of studies screened and provide reasons for study exclusion. | Figure 1 |
| 10 | Rationale for appraisal | Describe the rationale and approach used to appraise the included studies or selected findings. | Main manuscript pp.5-6, Supplementary File pp.2-3 |
| 11 | Appraisal items | State the tools, frameworks and criteria used to appraise the studies or selected findings. | Main manuscript pp.5-6, Supplementary File pp.2-3 |
| 12 | Appraisal process | Indicate whether the appraisal was conducted independently by more than one reviewer and if consensus was required. | Main manuscript pp.5-6, Supplementary File pp.2-3 |
| 13 | Appraisal results | Present results of the quality assessment and indicate which articles, if any, were weighted/excluded based on the assessment and give the rationale. | Main manuscript p.9, Table 2 |
| 14 | Data extraction | Indicate which sections of the primary studies were analysed and how were the data extracted from the primary studies? | Main manuscript p.5, Supplementary File p.2 |
| 15 | Software | State the computer software used, if any. | Main manuscript p.5 |
| 16 | Number of reviewers | Identify who was involved in coding and analysis. | Main manuscript p.5, Supplementary File pp.1-2 |
| 17 | Coding | Describe the process for coding of data. | Main manuscript p.5, Supplementary File pp.1-2 |
| 18 | Study comparison | Describe how were comparisons made within and across studies. | Main manuscript pp.5-6, Supplementary File pp.1-2 |
| 19 | Derivation of themes | Explain whether the process of deriving the themes or constructs was inductive or deductive. | Main manuscript p.5, Supplementary File pp.1-2 |
| 20 | Quotations | Provide quotations from the primary studies to illustrate themes/constructs and identify whether the quotations were participant quotations of the author’s interpretation. | Supplementary Table S16 |
| 21 | Synthesis output | Present rich, compelling and useful results that go beyond a summary of the primary studies. | Main manuscript pp. 9-21, Tables 3-4 |

Table S3: Medline (Ovid) Search Strategy

| Search Terms | Qualitative Filter Information |
| --- | --- |
| 1 exp Lung Neoplasms/  2 Lung Cancer*.mp.  3 Lung Neoplasm*.mp.  4 Lung Carcinoma*.mp  5 Lung Malignanc*.mp.  6 Bronchogenic Carcinoma*.mp.  7 1 or 2 or 3 or 4 or 5 or 6  8 exp Exercise/  9 Exercise*.mp.  10 Physical activit*.mp.  11 exp Rehabilitation/  12 Rehabilitation*.mp.  13 exp Exercise Movement Techniques/  14 exp Exercise Therapy/  15 exp Hydrotherapy/  16 Physiotherap*.mp.  17 Physical Therap*.mp.  18 exp Physical Therapy Modalities/  19 Tai Ji/  20 Tai Chi.mp.  21 Yoga.mp.  22 (aerobic or isometric or eccentric or stretching or treadmill or running or jogging or swimming or "strength training" or "weight training" or "weight lifting" or cycling).mp.  23 circuit-based exercise/ or endurance training/ or high-intensity interval training/ or plyometric exercise/ or resistance training/  24 Endurance Train*.mp.  25 High Intensity Interval Train*.mp.  26 Resistance Train*.mp.  27 Pulmonary Rehab*.mp.  28. exp Preoperative exercise/ or exp Telerehabilitation/ or exp walking/ or exp bicyc*/  29. 8 or 9 or 10 or 11 or 12 or 13 or 14 or 15 or 16 or 17 or 18 or 19 or 20 or 21 or 22 or 23 or 24 or 25 or 26 or 27 or 28  30 7 and 29  31 ((("semi-structured" or semistructured or unstructured or informal or "in-depth" or indepth or "face-to-face" or structured or guide) adj3 (interview* or discussion* or questionnaire*)) or (focus group* or qualitative or ethnograph* or fieldwork or "field work" or "key informant")).ti,ab. or interviews as topic/ or focus groups/ or narration/ or qualitative research/  32 30 and 31  33 (qualitative adj3 synthes*).mp.  34 (mixed adj3 method*).mp.  35 (multiple adj3 method).mp.  36 multimethod*.mp.  37 interview*.mp.  38 33 or 34 or 35 or 36 or 37  39 30 and 38  40 32 or 39 | Utilises the UTHealth Medline Qualitative Search Filter[19, 20] |

Table S4: EMBASE (Ovid) Search Strategy

| Search Terms | Qualitative Filter Information |
| --- | --- |
| 1, exp lung cancer/  3, Lung Neoplasm*.mp.  4, Lung Carcinoma*.mp.  5, Lung Malignanc*.mp.  6, Bronchiogenic Carcinoma*.mp.  7, 1 or 2 or 3 or 5 or 6  8, exp exercise/  9, Exercise*.mp.  10, exp physical activity/  11, Physical Activit*.mp.  12, exp rehabilitation/  13, Rehabilitation*.mp.  14, exp hydrotherapy/  15, Hydrotherapy.mp.  16, exp physiotherapy/  17, Physiotherap*.mp.  18, exp Tai Chi/  19, Tai Chi.mp.  20, exp yoga/,  21, Yoga.mp.  22, (aerobic or isometric or eccentric or stretching or treadmill or running or jogging or swimming or "strength training" or "weight training" or "weight lifting" or cycling).mp.  23, exp endurance training/  24, exp circuit training/  25, exp high intensity interval training/  26, exp plyometrics/  27, exp resistance training/  28, exp Cycling/ or exp Walking/  29, 8 or 9 or 10 or 11 or 12 or 13 or 14 or 15 or 16 or 17 or 18 or 19 or 20 or 21 or 23 or 24 or 25 or 26 or 27 or 28  30, 7 and 29  31, (("semi-structured" or semistructured or unstructured or informal or "in-depth" or indepth or "face-to-face" or structured or guide) adj3 (interview* or discussion* or questionnaire*)).ti,ab. or (focus group* or qualitative or ethnograph* or fieldwork or "field work" or "key informant").tw,kw. or qualitative research/  32, 30 and 31  33, (qualitative adj3 synthes*).mp.  34, (mixed adj3 method*).mp.  35, (multiple adj3 method*).mp.  36, multimethod*.mp.  37, interview*.mp.  38, 33 or 34 or 35 or 36 or 37  39, 30 and 38  40, 32 or 39 | Utilises a version of the UTHealth Medline Qualitative Search Filter adapted for EMBASE (Ovid)[19] |

Table S5: PsycINFO (Ovid) Search Strategy

| Search Terms | Qualitative Filter Information |
| --- | --- |
| 1., (lung cancer* or lung neoplasm* or lung malignanc* or lung carcinoma* or bronchogenic carcinoma*).mp.  2., exp exercise/  3., Exercise*.mp.  4., exp physical activity/  5., Physical Activit*.mp.  6., exp rehabilitation/  7., Rehabilitation*.mp.  8., Hydrotherapy.mp.  9., Physiotherap*.mp.  10., Physical Therap*.mp.  11., (Tai Chi or Tai Ji).mp.  12., Yoga.mp.  13., exp Walking/  14., (aerobic or isometric or eccentric or stretching or treadmill or running or jogging or swimming or "strength training" or "weight training" or "weight lifting" or cycling).mp.  15., 2 or 3 or 4 or 5 or 6 or 7 or 8 or 9 or 10 or 11 or 12 or 13 or 14  16., 1 and 15  17., (((("semi-structured" or semistructured or unstructured or informal or "in-depth" or indepth or "face-to-face" or structured or guide or guides) adj3 (interview* or discussion* or questionnaire*)) or (focus group* or qualitative or ethnograph* or fieldwork or "field work" or "key informant")).ti,ab,id. or exp qualitative research/ or exp interviews/ or exp group discussion/ or qualitative study.md.) not "Literature Review".md.  18., 16 and 17  19., ((qualitative adj3 synthes*) or (mixed adj3 method*) or (multiple adj3 method*) or multimethod* or interview*).mp.  20., 16 and 19  21., 18 or 20 | Utilises the UTHealth search filter for PsycINFO[19] |

Table S6: Scopus Search Strategy

| Search Terms | Qualitative Filter Information |
| --- | --- |
| TITLE-ABS-KEY ( ( "lung cancer*" OR "lung neoplasm*" OR "lung malignanc*" OR "lung carcinoma*" OR "bronchogenic carcinoma" ) AND ( exercise* OR "physical activit*" OR rehabilitation* OR telerehabilitation* OR hydrotherapy OR physiotherap* OR "physical therap*" OR aerobic OR isometric OR eccentric OR stretching OR walking OR treadmill OR running OR jogging OR swimming OR "strength training" OR "weight training" OR “weight lifting” OR cycling OR "tai chi" OR "yoga" OR "endurance training" OR "high intensity interval training" OR "resistance training" OR "pulmonary rehabilitation" ) ) AND KEY ( "qualitative research" OR interview OR "semi structured interview " OR "thematic analysis" OR " qualitative analysis" ) OR ( TITLE-ABS ( qualitative OR interview* OR "thematic analysis" OR themes OR "mixed method" OR "mixed methods" ) ) | Utilises a qualitative search filter recommended during the Cochrane QES training course |

Table S7: CINAHL (EBSCO) Search Strategy

| Search Terms | Qualitative Filter Information |
| --- | --- |
| 1. (MH "Lung Neoplasms+") OR ("lung cancer*" OR "lung neoplasm*" OR "lung malignanc*” OR “lung carcinoma*” OR bronchogenic carcinoma*") 2. (MH "Exercise+") OR (exercise* OR "circuit training" OR "endurance training" OR "high intensity interval training OR "resistance training" OR yoga OR "tai chi" OR hydrotherapy OR aerobic OR isometric OR eccentric OR stretching OR walking OR treadmill OR running OR jogging OR swimming OR "strength training" OR "weight training" OR “weight lifting” OR cycling OR walking) OR (MH "Physical Activity") OR ("Physical Activit*") OR (MH "Physical Therapists") OR (Physiotherap* OR "Physical Therap*") OR (MH "Rehabilitation+") OR (Rehab* OR Pulmonary Rehab*) 3. 1 AND 2   Limiter: Clinical Queries: Qualitative – Best Balance | Utilises a qualitative search filter inbuilt to the database |

Table S8: Web of Science Search Strategy

| Search Terms | Qualitative Filter Information |
| --- | --- |
| TS=("lung cancer*" OR "lung neoplasm*" OR "lung malignanc*" OR “lung carcinoma*” OR "bronchogenic carcinoma*") AND TS=(exercise* OR "physical activit*" OR rehabilitation* OR hydrotherapy OR physiotherap* OR "physical therap*" OR "tai chi" OR yoga OR "endurance training" OR "high intensity interval training" OR "resistance training" OR "pulmonary rehab* OR aerobic OR isometric OR eccentric OR stretching OR walking OR treadmill OR running OR jogging OR swimming OR "strength training" OR "weight training" OR “weight lifting” OR cycling OR walking ") AND TS=("Qualitative research" OR interview* OR "semi structured interview" OR "thematic analysis" OR "qualitative analysis" OR theme OR "mixed method*")Limiter: Clinical Queries: Qualitative – Best Balance | Utilises a qualitative search filter inbuilt to the database |

Table S9: PEDro Search Strategy

| Search Terms | Qualitative Filter Information |
| --- | --- |
| “Lung cancer exercise” | N/A |

Table S10: Cochrane Library Search Strategy

| Search Terms | Qualitative Filter Information |
| --- | --- |
| #1 MeSH Descriptor: [Lung Neoplasms] 1 tree exploded  #2 “Lung Cancer” OR “Lung Neoplasm*” OR “Lung Malignanc*” OR “Lung Carcinoma*” OR “Bronchogenic Carcinoma”  #3 #1 OR #2 #4 MeSH Descriptor: [Exercise] 1 tree exploded  #5 MeSH Descriptor: [Rehabilitation] 1 tree exploded  #6 Exercise* OR “Physical Activit*” OR Rehabilitation* OR Physiotherap* OR “Physical Therap*” OR ”Tai Chi”OR Yoga OR Hydrotherapy OR aerobic OR isometric OR eccentric OR stretching OR walking OR treadmill OR running OR jogging OR swimming OR "strength training" OR "weight training" OR “weight lifting” OR cycling OR walking  #7 #4 OR #5 OR #6  #8 MeSH Descriptor: [Qualitative Research] explode all trees  #9 Qualitative OR Interview* OR Focus Group* OR Theme* OR Mixed Method* OR Multimethod*  #10 #8 OR #9  #11 #3 AND #7 AND #10 | N/A |

Table S11: Grey Literature Search Strategies

| Repository | Search Strategy |
| --- | --- |
| Google Scholar | allintitle: lung cancer "physical activity" OR exercise |
| ProQuest Dissertations & Theses Global | ab("lung cancer") AND ab(("physical activity" OR exercise)) |
| EBSCO Open Dissertations | (“lung cancer”) AND (“physical activity” or “exercise”) |
| EThOS | "Lung cancer" AND ("physical activity" OR "exercise") |
| Open Access Theses and Dissertations | ("lung cancer") AND abstract:(exercise) OR abstract:("physical activity") |

Table S12: Characteristics of included studies

| (#) | First Author, Year, Country | Summary of Study Aims | Study Design | Qualitative Data Collection | Qualitative Data Analysis |
| --- | --- | --- | --- | --- | --- |
| (1) | **Adamsen, 2012, Denmark** | Explore the feasibility and experienced health benefits of participation in the program from the patients’ perspective | Qualitative feasibility study | Semi-structured interviews and one semi-structured focus group | Thematic analysis |
| (2) | **Collaco, 2022, United Kingdom** | Explore patients and clinicians’ experiences of the program | Qualitative study | Semi-structured interviews | Thematic analysis |
| (3) | **Conte, 2020, Canada** | Assess the feasibility of the program and its evaluation, pilot the collection of PROs, identify outcomes that may be positively influenced, and inform program modifications | Observational pilot study | MYCaW, internally developed questionnaires, and semi-structured interviews | Data was pooled and assessed for common and unique themes |
| (4) | **Crandall, 2016, Scotland*** | Test the feasibility and acceptability of the program | Feasibility study | Semi-structured interviews | Thematic framework analysis |
| (5) | **de Leeuwerk, 2022, The Netherlands** | Collect quantitative and qualitative data to investigate the feasibility of the program | Mixed-methods feasibility study | Semi-structured interviews | Thematic analysis |
| (6) | **Edbrooke, 2019, Australia** | Characterise the views and experiences of participants of the program and understand factors impacting acceptability and feasibility | Qualitative study | Semi-structured interviews | Conventional content analysis |
| (7) | **Ester, 2021, Canada** | Assess the feasibility of the program | Mixed-methods study | Semi-structured interviews | Interpretive description |
| (8) | **Finley, 2020, United States** | Explore the feasibility, acceptability and perceived utility of the program | Feasibility study | Semi-structured interviews | Content analysis |
| (9) | **Groen, 2017, The Netherlands** | Evaluate the program’s feasibility and usability | Feasibility study | Focus group and open-ended survey questions | Analysis and integration, topics included if raised by >2 participants |
| (10) | **Henshall, 2020, United Kingdom** | Design, develop and test the program | Usability study | Think-aloud tasks and semi-structured interviews | Framework analysis |
| (11) | **Hoffman, 2014, United States** | Uncover an in-depth understanding of the highest priority unmet supportive care needs of patients | Qualitative study | Focus group | Content analysis |
| (12) | **Hoffman, 2017, United States** | Provide further details regarding the acceptability of the program | Qualitative study | Open-ended questionnaire | Content analysis |
| (13) | **Ji, 2022, China** | Provide qualitative evidence regarding the acceptability of, enablers of, and barriers to the program | Qualitative study | Semi-structured interviews | Thematic analysis |
| (14) | **Kadiri, 2019, United Kingdom** | Develop a program and test its feasibility and acceptability | Cohort study | Semi-structured interviews | Content analysis |
| (15) | **Langballe, 2023, Denmark** | Determine the feasibility and acceptability of the program to identify needed adjustments of program components and procedures and evaluate outcomes | One-armed, intervention-only feasibility study | Semi-structured interviews | Deductive coding and summarising according to the seven constructs of the TFA |
| (16) | **McDonnell, 2020, United States** | Evaluate the feasibility and preliminary effects of the program | Prospective pilot study | Interviews | Thematic analysis |
| (17) | **Missel, 2015, Denmark** | Explore participant experiences of the program from a longitudinal perspective according to patient motivation and perceived benefits and barriers | Qualitative longitudinal feasibility study | Semi-structured interviews (collected at 3 time points) | Ricoeur’s Theory of Interpretation |
| (18) | **Missel, 2019, Denmark** | Explore the lived experiences and social benefits of the program | Qualitative study | Interviews (collected at 3 time points) | Ricoeur’s Theory of Interpretation |
| (19) | **Payne, 2018, Northern Ireland** | Explore the experiences and perceptions of participants and clinicians involved in the program regarding feasibility, acceptability, benefits, and burdens | Qualitative study | Semi-structured interviews | Thematic analysis |
| (20) | **Rewar, 2018, United States** | Explore the benefits of and participation satisfaction with the program, and inform future directions (secondary aim) | Pilot feasibility study | Surveys and focus groups | Analysis based on consensus between participants |
| (21) | **Shukla, 2020, Australia** | Explore the views and experiences of participants in the program and usual care group (secondary aim) | Pilot randomized controlled trial | Semi-structured interviews | Thematic analysis |
| (22) | **Somayaji, 2019, United States** | Explore participants’ perspectives on making behavioural changes in the context of the program | Qualitative study | Semi-structured interviews | Inductive content analysis |
| (23) | **Sun, 2020, United States** | Determine the barriers and facilitators of adherence to the program | Qualitative study | Free-text data from PT/OT notes written after sessions | Content analysis |
| (24) | **Timmerman, 2017, The Netherlands*** | Evaluate the feasibility of the program | Mixed-methods feasibility study | Focus groups and questionnaires | Notes from the focus group were combined with the questionnaire to highlight the most important aspects |

* data from the evaluation phase of a multi-phase program development and evaluation study

**Abbreviations**: MYCaW = Measure Yourself Concerns and Wellbeing outcome measure; PT = physiotherapist; OT = occupational therapist; TFA = theoretical framework of acceptability

**Table S13: Characteristics of patients with lung cancer participating in qualitative data collection**

| (#) | First Author, Year, Country | n | Sex  M:F | Age  mean +/- SD | Lung Cancer Type, n (%) | Lung Cancer Stage, n (%) | Primary Lung Cancer Population | Cancer treatment(s) planned and/or received n (%) |
| --- | --- | --- | --- | --- | --- | --- | --- | --- |
| (1) | **Adamsen, 2012, Denmark** | 15 | 7:8 | 66 (45-80) ^A^ | NSCLC 13 (87)  SCLC 2 (13) | IIIA 4 (31)  IIIB 7 (54)  IV 2 (15)  ES 2 (13) | Inoperable/ Advanced | Chemotherapy 15 (100)  Radiotherapy 10 (67) |
| (2) | **Collaco, 2022, United Kingdom** | 17 | 7:10 | 40-49: 1 (6)  50-59: 0 (0)  60-69: 4 (24)  70-79: 9 (53)  80-89: 3 (17) ^B^ | NSCLC 12 (70)  SCLC 1 (6)  Missing 4 (24) | NR | Operable | Surgery 17 (100)  Chemotherapy 2 (12) |
| (3) | **Conte, 2020, Canada** | 8 | 1:7 | 59.6 +/- 9.7 | NR | NR | Mixed | Surgery 2 (25)  Chemotherapy 2 (25)  Radiotherapy 3 (38)  Immunotherapy/Targeted Therapy 6 (75) |
| (4) | **Crandall, 2016, Scotland*** | 9/14 ^C^ | 5:9 | 68.3 +/- 9.57 | NR | NR | Predominantly Operable | Surgery 12 (86)  Adjuvant/neoadjuvant/others NR |
| (5) | **de Leeuwerk, 2022, The Netherlands** | 6/41 ^C,D^ | 25:16 | 68 [60-73] ^E^ | NR | I 7 (17)  II 8 (20)  III 6 (15)  IV 18 (44)  Schwannoma 2 (5) | Operable | Surgery 41 (100)  Neoadjuvant chemotherapy 3 (7)  Neoadjuvant chemoradiotherapy 9 (22)  Neoadjuvant immunotherapy 1 (2)  Neoadjuvant hormone therapy 1 (2)  Adjuvant chemoradiotherapy 2 (5)  Adjuvant chemotherapy 3 (7)  Adjuvant radiotherapy 1 (2)  Adjuvant hormone therapy 1 (2) |
| (6) | **Edbrooke, 2019, Australia** | 25 | 13:12 | 66.5 +/- 12.5 | NSCLC 25 (100) | IA 1 (4)  IB 1 (4)  IIIA 7 (28)  IIIB 2 (8)  IV 11 (44) Recurrent 3 (12) | Inoperable | Chemotherapy 4 (16)  Radiotherapy 7 (28)  Chemotherapy and Radiotherapy 10 (40)  Targeted Therapy 2 (8)  Systemic Clinical Trial Therapy 2 (8) ^F^ |
| (7) | **Ester, 2021, Canada** | 10 | 4:6 | 64.4 +/- 10.3 | NSCLC 10 (100) | IV 10 (100) | Inoperable/ Advanced | Targeted Therapy 6 (60)  Immunotherapy 2 (20)  Targeted therapy and chemotherapy 1 (10)  N/A 1 (10) ^F^ |
| (8) | **Finley, 2020, United States** | 16/28 ^C^ | 12:16 | 67.3 +/- 10.6 | NR | NR | Operable | Surgery 16 (100)  Adjuvant/neoadjuvant/others NR |
| (9) | **Groen, 2017, The Netherlands** | 5/34 ^C^ | 18:16 | 59.6 +/- 8.4 | NSCLC 34 (100) | I 13 (38)  II 5 (15)  III 16 (47) | Mixed | Surgery 12 (35)  Surgery and Chemotherapy 3 (9)  Surgery and Chemoradiotherapy 2 (6)  Chemoradiotherapy 10 (29)  Radiotherapy 7 (21) |
| (10) | **Henshall, 2020, United Kingdom** | 6 | 2:4 | 51-60: 1 (33)  61-70: 1 (33)  >70: 4 (67) ^B^ | NSCLC 6 (100) | NR | NR | NR |
| (11) | **Hoffman, 2014, United States** | 6 | 2:4 | 63.8 +/- 6.6 ^F^ | NSCLC 6 (100) | IB 1 (17)  IIA 1 (17)  IIB 2 (33)  IIIA 2 (3) | Operable | Surgery 6 (100)  Chemotherapy and radiotherapy 3 (50)  Chemotherapy 2 (33) |
| (12) | **Hoffman, 2017, United States** | 37 | 17:20 | 67.4 +/- 9.7 | NSCLC 37 (100) | I or II 29 (78)  III or IV 6 (16)  Indeterminate 2 (6) | Operable | Surgery 37 (100)  Adjuvant/neoadjuvant/others NR |
| (13) | **Ji, 2022, China** | 14 | 7:7 | 62.6 +/- 8.5 ^F^ | NR | NR | Operable | Surgery 14 (100)  Chemotherapy 2 (14) |
| (14) | **Kadiri, 2019, United Kingdom** | 13/31 ^C^ | NR | 64 +/- 12 | NSCLC 17 (55)  Other lung 7 (23)  Metastasis 1 (3)  Benign 6 (19) | NR | Operable | Surgery 31 (100)  Adjuvant/neoadjuvant/others NR |
| (15) | **Langballe, 2023, Denmark** | 11 ^G^ | 7:4 | 73 +/- 6 | NSCLC 11 (100) | I-IIIA 2 (18)  IIIB-IV 9 (81) | NR ^H^ | NR |
| (16) | **McDonnell, 2020, United States** | 20/26 ^C^ | 16:10 | 66.5 +/- 5.5 | NSCLC 20 (100) | NR | NR | NR |
| (17) | **Missel, 2015, Denmark** | 19 | 8:11 | 63 [48-75] ^E^ | NSCLC 19 (100) | IA 5 (26)  IB 1 (5)  IIA 9 (47)  IIB 2 (11)  III 2 (11) | Operable | Surgery 19 (100)  Adjuvant chemotherapy 8 (42) |
| (18) | **Missel, 2019, Denmark** | 18 | 8:10 | 67 [36-79] ^E^ | NSCLC 18 (100) | NR | Operable | Surgery 18 (100)  Adjuvant chemotherapy 8 (44) |
| (19) | **Payne, 2018, Northern Ireland** | 8 | 5:3 | 60 (46-68) ^A^ | NSCLC 8 (100) | IIIB 2 (25) IV 6 (75) | Inoperable/ Advanced | Chemotherapy 5 (63)  Targeted Therapy 2 (26)  Chemotherapy, targeted therapy and radiotherapy 1 (13) |
| (20) | **Rewar, 2018, United States** | 13 | 1:12 | 66.5 +/- 8.9 | NSCLC 11 (85)  SCLC 2 (15) | I 3 (2)  II 2 (15)  IV 6 (46)  LS 1 (8)  ES 1 (8) | Mixed | Surgery 7 (54)  Radiotherapy 7 (54)  Chemotherapy 8 (61)  Targeted therapy 3 (23)  Immunotherapy 1 (8) |
| (21) | **Shukla, 2020, Australia** | 11 | 5:6 | 66.5 +/- 8.6 | NSCLC 11 (100) | IA 5 (50)  IB 1 (10) IIA 1 (10)  IIB 1 (10)  IIIA 2 (20)  Missing 1 (10) | Operable | Surgery 11 (100)  Adjuvant/neoadjuvant/others NR |
| (22) | **Somayaji, 2019, United States** | 22 | 7:15 | 66 (40-75) ^A^ | NSCLC 22 (100) | I 11 (50)  II 6 (27)  III 5 (23) | Operable | Surgery 22 (100)  Adjuvant/neoadjuvant/others NR |
| (23) | **Sun, 2020, United States** | 18 | 9:9 | 74.0 (68-83) ^A^ | NR | NR | Operable | Surgery 18 (100)  Adjuvant/neoadjuvant/others NR |
| (24) | **Timmerman, 2017, The Netherlands*** | 12 | 8:4 | 60 [55-66] ^E^ | NSCLC 12 (100) | NR | Predominantly Operable | Surgery 10 (83)  Neoadjuvant treatment 2 (17%) (type NR)  Adjuvant treatment 1 (8%) (type NR) |

**Abbreviations:** ES = SCLC extensive stage; F = female; IQR = interquartile range; LS = SCLC limited stage; M = male; n= number; SCLC = small cell lung cancer; SD = standard deviation; NR = not reported; NSCLC = non-small cell lung cancer.

* data from the evaluation phase of a multi-phase program development and evaluation study

^A^ mean (range)

^B^ range, n (%)

^C^ mixed methods study that included a sub-set of participants in the qualitative element but did not report their demographics separately from the primary cohort

^D^ study included a mixed cancer cohort but did not report the demographics of patients with lung cancer separately. 21/41 (51%) of the sample had lung cancer, and 5/6 of those interviewed appeared to have lung cancer.

^E^ median [IQR]

^F^ calculated or aggregated by review authors

^G^ only 6 (55%) participated in the exercise component of the multi-modal program

^H^ reported treatment intent: curative n=1 (9%), palliative n=9 (82%).

**Table S14: Characteristics of non-patients participating in qualitative data collection**

| (#) | First Author, Year, Country | Participant group(s)  n (%) | Sex M:F | Age  mean +/-SD |
| --- | --- | --- | --- | --- |
| (2) | **Collaco, 2022, United Kingdom** | Clinicians (n=8)  Nurse 4 (50)  Advanced Therapist Practitioner 1 (12.5)  Surgeon 1 (12.5)  Respiratory 1 (12.5)  Physician 1 (12.5) | 1:7 | NR |
| (3) | **Conte, 2020, Canada** | Clinicians (n=1)  Program facilitator 1 (100)  Others (n=2)  Survivor advocates 2 (100) | NR | NR |
| (4) | **Crandall, 2016, Scotland *** | Clinicians (n=4)  Physiotherapist NR  Nurse NR | NR | NR |
| (15) | **Langballe, 2023, Denmark** | Clinicians (n=NR)  Nurse navigators NR  Physiotherapists NR | NR | NR |
| (16) | **McDonnell, 2020, United States** | Family members (n=19/23) ^A^  Relationship NR | 13:10 | 60.2 +/- 14.1 |
| (19) | **Payne, 2018, Northern Ireland** | Clinicians (n=6)  Clinical trials nurse 4 (67)  Clinical oncologist 1 (17)  Medical oncologist 1 (17) | 0:6 | 30–39: 2 (33)  40–49: 3 (50)  50–65: 1 (17) ^B^ |
| (20) | **Rewar, 2018, United States** | Caregivers (n=9)  Friend 3 (33)  Spouse 5 (56)  Family member 2 (22) | 4:5 | 62.4 +/- 18.3 |
| (24) | **Timmerman, 2017, The Netherlands *** | Clinicians (n=2)  Physiotherapist 2 (100%) | NR | NR |

**Abbreviations:** F = female; M = male; n= number; NR = not reported; SD = standard deviation

* data from the evaluation phase of a multi-phase program development and evaluation study

^A^ mixed methods study that included a sub-set of participants in the qualitative element but did not report their demographics separately from the primary cohort

^B^ range, n (%)

**Table S15: Characteristics of included programs (based on the TIDieR checklist[21])***

|  |  | **Program:** | | | | | | | **Exercise:** | | | |
| --- | --- | --- | --- | --- | --- | --- | --- | --- | --- | --- | --- | --- |
| **(#)** | **First Author, Year** | **Timing relative to primary cancer treatment** | **Duration**  **(weeks)** | **Setting** | **Provider** | **Delivery mode** | **Sessions per week** | **Session time (minutes)** | **Types** | **Intensity** | **Individualisation** | **Progression** |
| **(1)** | Adamsen, 2012 | During | 6 | Fitness facility within a hospital | Research PT and a clinical nurse specialist | Supervised group | 2 | 90 | Warm-up (stationary cycling), resistance training (leg press, chest press, pull down, abdominal crunch, lower back, and knee extension machines), interval cardiovascular exercise (stationary cycling), stretching and relaxation | Resistance training: 5-8 repetitions of 80-90% 1RM  CVS training: 70-95% max HR (15-20 on Borg Scale) | Adjusted based on target HR and 1RM | Resistance training was progressed based on weekly 1RM testing. Aerobic progression NR. |
|  |  |  |  | Home-based |  | Unsupervised | 3 | 20 (gait training x 2 daily) | Gait training (detail NR), respiration and relaxation | NR |  |  |
| **(2)** | Collaco, 2022 | Before and after | 8 ^A^ | Hospital and/or home | NR | Supervised group and/or optional unsupervised home exercise | 2 | NR | NR | NR | Personalised based on participant requirements | NR |
| **(3)** | Conte, 2020 | Before, during or after | 6 | Integrative cancer centre | Certified yoga therapist, clinic director and two survivor advocates | Supervised group | 1 | 60 | Gentle yoga (chair, standing and some gentle floor work) and breath practice | ‘Gentle’ (further detail NR) | NR | NR |
| **(4)** | Crandall, 2016 | Before | 3 | Centre-based (location NR) | PT | Supervised group | 2 | 60 | ‘Pulmonary rehabilitation’ circuit of aerobic (walking or running on a treadmill, rowing machine or other floor-based exercise) and resistance exercise (hand weights and resistance bands). | Moderate (detail NR) | Goals were set individually, and participants were prescribed exercises based on ability and preference | NR |
|  |  |  |  | Home-based |  | Unsupervised | >3 | NR | Walking (detail NR) | NR (advice to aim for 7000-10,000 steps per day) |  |  |
| **(5)** | de Leeuwerk, 2022 | After^B^ | 6 | Home-based | PT | Unsupervised, supported by 1x initial inpatient consultation, ‘ad-hoc’ PT contact and a wearable accelerometer and web-based app | NR | NR | NR | NR | Patients received ‘personalised rehabilitation recommendations’ | NR (PTs monitored via web application weekly, if activity levels decreased or no data, PT contacted the patient) |
| **(6)** | Edbrooke, 2019 | During | 8 | Home-based | PT | Unsupervised, monitored by telephone or F2F check-in appointment | >2 | >10 | Aerobic (walking, cycling, or swimming) and resistance exercise (sit-to-stand, squats, heel raises, wall press, and upper limb free weights) | Aerobic: moderate (4-6 on Borg dyspnoea scale)  Resistance: 80% of 10RM in 2-3 sets | Endurance training individualised to participant preference and ability | Weekly progressions when participants had achieved the previous week’s goals and working below target RPE |
| **(7)** | Ester, 2021 | During and/or after | 12-14 | Centre-based (location NR) | Clinical EP | Supervised group | 1-2 | NR | Aerobic, resistance and flexibility exercise (detail NR) | Light-to-moderate (detail NR) | Sessions were individually tailored | Program progressed at 4,6, 8 and 10 weeks (additional resistance/sets/repetitions or exercise progression) |
|  |  |  |  | Home-based |  | Unsupervised | 2-4 | 10-30 | Aerobic (i.e., walking) and resistance exercise (using body weight and an exercise band or ball) |  |  |  |
| **(8)** | Finley, 2020 | Before | Varied ^C^ | Home-based | Surgeon | Unsupervised | 5 | 30 | Aerobic exercise (any activity, suggestions included walking, jogging, stair climbing, upper body ergometer or stationary bicycle) | Moderate (detail NR) | Participants were encouraged to choose any form of exercise | NR |
| **(9)** | Groen, 2017 | During and/or after | Varied ^D^ | Home-based | NR | Unsupervised, delivered via an interactive online portal | NR | NR | Non-specific advice to increase general levels of physical activity based on a set of questionnaires | NR | Advice was tailored based on questionnaires and possible contraindications | NR |
| **(10)** | Henshall, 2020 | After | 2 | Home-based | NR | Unsupervised, delivered via a mobile app | NR | NR | Exercises were generated by inputting symptoms and preferences (specific exercises NR) | NR | Exercises were tailored and generated by an algorithm based on symptoms and preferences | NR |
| **(11)** | Hoffman, 2014 | After | 16 | Home-based | Nurses | Unsupervised, monitored by telephone or F2F check-in appointment | 5 | Varied ^E^ | Continuous walking and balance exercises using Wii Fit | Light (<3.0 metabolic equivalents) | Balance exercises were predetermined& walking speed determined by perceived self-efficacy | Walking duration was progressed weekly based on perceived-self-efficacy |
| **(12)** | Hoffman, 2017 | After | 6 | Home-based | Nurses | Unsupervised, supported by 1x F2F consult for set-up, monitored by telephone or F2F check-in appointment | 5 | Varied ^E^ | Continuous walking and balance exercise using Wii Fit | Light (<3.0 metabolic equivalents) | Balance exercises were predetermined & walking speed determined by perceived self-efficacy | Walking duration was progressed weekly based on perceived-self-efficacy |
| **(13)** | Ji, 2022 | After | 4 | Home-based | Nurses | Unsupervised, monitored by telephone | 5 | 50 | Walking (detail NR) | Low-to-moderate (detail NR) | NR | NR |
| **(14)** | Kadiri, 2019 | Before and after | NR | Home-based | Researchers | Unsupervised, delivered via an app | NR | >30 | Upper and lower limb aerobic and strength exercise based on the ‘Rehabilitation for Operated lung Cancer surgery program.’ Further detail NR. | Target HR >60% max | NR | NR |
| **(15)** | Langballe, 2023 | Before and during | 12 | Hospital (first 8 weeks) | PT | Supervised small group or individual | 2 | 40 | Aerobic exercise such as walking or cycling; and muscle strength and endurance (including pull-to-chest, sit-to-stand, shoulder press, and abdominal crunch) | Aerobic: 14-15 on BORG scale  Strength and endurance: three sets of 15 repetitions with elastic bands ‘of various strengths’ | Tailored through manualised criteria for progression or regression of the intensity level | Aerobic:  At end of week 5 if required to obtain >14 on BORG scale  Strength and endurance: continuously when patients able to perform >15 repetitions in the last set (increased band resistance) |
|  |  |  |  | Home-based (final 4 weeks) |  | Unsupervised, supported by online videos and telephone guidance |  |  |  |  |  |  |
| **(16)** | McDonnell, 2020 ^F^ | After | 8 | Commun-ity studio adjacent to major university | Advanced practice nurse and a board-certified psychiatrist | Supervised group | 1 | 120 | Mindful hatha yoga (individual and partner poses) | Gentle/low intensity (detail NR) | Participants could choose between level 1 (sitting poses only) or level 2 (sitting, standing and floor poses). | NR |
|  |  |  |  | Home-based |  | Unsupervised | 7 | NR |  |  |  |  |
| **(17, 18)** | Missel, 2015 and 2019 ^G^ | After | 12 | Commun-ity based training centre within a cancer-specific health service | PT and a cancer nurse specialist | Supervised group | 2 | 60 | Cardiovascular (ergometer bike), strength (machine-based leg press, chest press, leg extension, pull to chest and pull down) and breathing, stretching & relaxation exercises | CVS: started at low intensity (~50%-60% max HR), increased to moderate-to-vigorous (70-90% of max HR)  Strength: 60-80% of 1RM (load was progressively increased and number of repetitions decreased throughout each session) | All interventions were individually tailored | Cardiovascular intensity progressed throughout the program. Strength exercise progressed every second week. |
| **(19)** | Payne, 2018 | During | 6 | Home-based | Researchers | Unsupervised, monitored by telephone or F2F check-in appointment | Varied | Varied | Cardiovascular (walking program) and resistance exercise (wall-press, sit-to-stand, arm raise, leg raise, arm curls and assisted knee bands using bands) | Moderate intensity (detail NR) | Individualised based on participants’ readiness to change and attitude to the program | Daily step goals were adjusted based on activity and barriers/enablers, and resistance exercises were introduced in a ‘stepped’ manner. |
| **(20)** | Rewar, 2018 ^F^ | During and/or after | 8 | Cancer rehabilitation clinic within a University | NR | Supervised individual | 1 | 60-120 | Aerobic exercise warm-up (detail NR), resistance exercise (concentric and eccentric scapular retraction, scapular elevation, elbow flexion, elbow extension, external rotation, abduction in scapular plane, leg extension, leg flexion and hip extension/abduction), stretching | Aerobic warm-up: low intensity  Resistance exercise: 40-60% of 1RM and no greater than 13 on Borg Scale | All participants were prescribed the same exercises. Resistance exercise load was based on 1RM testing. | Yoga was progressed every 2-weeks based on a protocol; and resistance exercise was progressed in pre-defined increments based on 1RM and dyspnoea. |
|  |  |  |  | University Cancer rehab clinic |  | Supervised | 1 | 60 | Warm up (breathing/stretching or aerobic exercise) and yoga | Low intensity |  |  |
|  |  |  |  | Home-based |  | Unsupervised | NR | NR | Breathing, postural correction and core resistance exercise (e.g., planking, push ups, sit-to-stands) | NR |  |  |
| **(21)** | Shukla, 2020 | Before | Varied  ^H^ | Hospital | PT or PT AHA | Supervised individual | 3 | 45 | Aerobic (stationary bike or treadmill) and resistance exercise (free weight or resistance machine for major upper and lower limb muscle groups) | Aerobic: 60-80% predicted maximum based on ISWT  Resistance: based on individual 1RM. | Exercises were tailored to the individual participant | All exercises were progressed as appropriate. |
|  |  |  |  | Home-based |  | Unsupervised, monitored via phone call | 2 | 30-45 | Aerobic (home stationary bike or outdoor walking) and resistance exercise (TheraBand). | Aerobic: 60-80% of maximal workload  Resistance: NR |  |  |
| **(22)** | Somayaji, 2019 | After | 8 | Home-based | Nurses | Unsupervised, monitored by telephone | NR | NR | NR (type of exercise based on ‘recommended guidelines’) | Variable (intensity based on ‘recommended guidelines’) | Recommendations personalised based on participants’ personal lifestyle behaviours | NR |
| **(23)** | Sun, 2020 ^F^ | Before and after | Varied  ^I^ | Home-based | PT and OT | Unsupervised, monitored by telephone or video call | NR | NR | Walking and lower extremity exercise (sit-to-stand, step up and down front, step up and down sideways, standing wall push). | Variable (based on participant functional status) | Exercise programs were ‘personalized’ | NR |
| **(24)** | Timmerman, 2017 | Before and after | 13-26 | Home-based | PT | Remotely supervised, supported/ monitored via an online module | NR | NR | Varied (prescribed by PT) | Variable (prescribed by PT) | Exercise program was ‘tailored’ and prescribed by the PTs | NR |

* Exercise component of programs only – i.e., additional components of multi-modal programs not reported here

**Abbreviations:** AHA = allied health assistant(s); CVS = cardiovascular system; EP = exercise physiologist(s); F2F = face-to-face’ HR = heart rate; NR = not reported; OT = occupational therapist(s); PT = physiotherapist(s); 1RM = one-repetition maximum.

^A^ preoperative program duration NR, postoperative duration 8 weeks

^B^ wearable activity tracking commenced 1 week prior to surgery for ‘exploration purposes’

^C^ mean 28.5 days

^D^ up to 4 months

^E^ between 5-30 minutes walking

^F^ caregivers/family members/loved ones also participated in program

^G^ programs were identical as per protocol and therefore reported as one

^H^ as many weeks as possible from commencement until surgery

^I^ at least 1 week before surgery until up to 4 weeks postoperatively

Table S16: Findings and supporting data

| # | Summarised review finding | Supporting data (first and second order) |
| --- | --- | --- |
| Theme 1: Components of exercise program design | |  |
| *Exercise prescription* | |  |
| 1 | Programs that prescribed exercise at an ‘achievable’ intensity and provided ‘controlled exposure’ to exercise supported participants in building exercise self-efficacy. Participants also appreciated provider-led exercise progression throughout the program. | “I’m very limited on my abilities to walk and breathe all at the same time, so the movements allowed me to enjoy some form of exercise without really feeling like I had to walk 10 miles or be able to twist into a pretzel, and yet still reap the benefits. So that was really good.” – Participant, Study 16 |
| 2 | Participants valued individualisation and tailoring, including individualised exercise prescription, goal setting, and education. Individualisation was seen as an enabler of exercise participation, and some participants gleaned additional motivation to participate in exercise by feeling individually cared for. " | “Facilitator: Programming components: instructor knowledge of cancer, personalized program, progression and modifications to exercise.” – Author interpretation, Study 20 |
| 3 | Walking was an accessible, simple and enjoyable form of exercise highly valued by participants. | “The majority of participants (n = 9, 64%) felt that walking was a suitable simple type of exercise or that walking was their preferred past exercise type…The convenience of walking was seen as another facilitator by all participants. They could perform their walking exercises in the area where they lived.”  - Author interpretation, Study 13 |
| 4 | Preferences regarding exercise types were variable, highlighting the importance of autonomy and providing various exercise options to allow for individual preferences. Variety was seen as an enabler of sustained exercise enjoyment and behaviour change, and some participants viewed programs that did not offer this flexibility as regimented and boring. | “Participants mentioned that pulmonary rehabilitation was not as strenuous as they thought it would be, liked that it included a variety of exercises and felt that they were able to try exercises they had not done before.” – Author interpretation, Study 4 |
| 5 | Some participants and providers of pre- and rehabilitation programs desired a longer program and/or more frequent sessions because they believed that longer programs were more beneficial. | “Whilst some participants were motivated to take part because of the short duration, others queried whether the study was of a sufficient length to fully determine the potential physical benefits of [the program]. “The study lasted 12 weeks.” – Author interpretation. “You know is that a long enough period for it?” – Participant, Study 19 |
| *Exercise setting* | | |
| 6 | Participants in group programs saw them as valuable and an essential source of motivation, socialisation, and support. One study discussed group size and reported that participants tended to prefer smaller group sizes to foster familiarity, intimacy, and closeness and reduce feelings of intimidation and overwhelm. Participants valued face-to-face supervision and opportunities for one-on-one support from program providers to ensure they completed prescribed exercises correctly. Some participants of centre-based programs/ desired an individualised home exercise program to support behaviour change and independent exercise outside supervised sessions. | “Training in a group is much better because you know that someone is keeping an eye out whether you come or not. It's a little more difficult when you have to do it on your own” - Participant. “I'm not good at working out at home. It helps when we nudge each other with [the research physiotherapist] and the others …” – Participant, Study 1 |
| 7 | Participants of home-based programs saw them as valuable. Many, especially participants with more advanced lung cancer, viewed home-based exercise as less burdensome than attending centre-based programs. | “Twelve of 14 respondents selected home as their preferred site of intervention for reasons including the time, costs and energy expended getting to hospital. Home also enabled a more flexible routine, allowing exercise to be balanced with rest or arranged around how they were feeling.” – Author interpretation, Study 6 “Yes (possible), but it’s a little bit too hard to get there every day…” – Participant, Study 6 |
| 8 | Participants of digital programs had mixed experiences, with some reporting drawbacks such as inadequate tailoring, flexibility, feedback, and support. | “During the focus group, this patient indicated a lack of interaction with the physiotherapist, insufficient tailoring of the exercises, and lack of insight in progression as most important reasons for dissatisfaction.” – Author interpretation, Study 24 |
| *Outcome measures* | | |
| 9 | Participants viewed participating in outcome measurement assessments as a way to improve motivation, resilience and self-efficacy by allowing participants to exceed their own expectations, demonstrating what is possible, and providing an objective measurement of their capacity and progress. | Probably the most important one was actually the original assessment. Um, I had been largely avoiding doing things cause I would immediately run out of breath. […] The assessment showed me that uh you can run out of breath and keep on going. Okay, and doing things, um, so that was probably the most important thing looking back, was getting started on doing things.” – Participant, Study 7 |
| 10 | Some participants desired provider feedback regarding their performance in outcome measurements and progress throughout the program. | “PROs were not often discussed during medical consultations, which disappointed some patients” – Author interpretation, Study 9 |
| 11 | Participants' views regarding the acceptability and utility of specific outcome measures were varied. In one study, participants and providers viewed physical outcome measures such as the Six-Minute Walk Test and the 60-second Sit-to-Stand Test as useful, relevant tools to monitor fitness and progress. Providers in another study reported that the Ekblom-Bak test was too burdensome for participants to complete. Participants of two studies found Patient Reported Outcomes (PROs) overly burdensome, cumbersome, and/or difficult to complete, whereas participants of another found them acceptable. | “Four participants described “the paperwork,” “answering questionnaires,” or “the phone surveys” as areas they liked least.” – Author interpretation, Study 12  “The 6MWT and STS functional outcome measures were of interest to participants for judging their own fitness levels at the start of the programme and assessing changes in fitness over time.” – Author interpretation. “Well, I think it’s motivated me …to get me started … when I wasn’t out of breath doing things and all, I felt good…even that [6MWT] and the [60STS].” – Participant, Study 19 |
| *Program timing* | | |
| 12 | Participants of some prehabilitation-only programs desired program continuation and support postoperatively to guide them to resume exercise and normal activities due to postoperative symptoms and physical inactivity. | “Participants also expressed a desire for ongoing follow-up and support from allied health professionals beyond the postoperative period, especially where they experience ongoing symptoms and were uncertain of the correct physical activity levels and timeframes to resume normal activities.” – Author interpretation, Study 21 |
| Theme 2: Providers of exercise programs | | |
| *Expertise* | | |
| 13 | Participants valued program providers’ expertise independent of their specialty/discipline, including expert education, guidance, monitoring, and exercise progression, regression, and modification. Support from a qualified expert improved confidence, safety, and trust. | The patients who participated in the training felt physically supported following surgery. They felt a sense of security that the physiotherapists and nurses were knowledgeable about their illness and exercise.” – Author interpretation, Study 17 |
| *Role* | | |
| 14 | Providers played a key role in supporting, encouraging, and educating participants to exercise and were seen as key instigators of health behaviour change by acting as important sources of extrinsic motivation and supporting participants to build exercise self-efficacy. | “The instructors and nurses are good at organizing, looking after you and motivating you to do a little more than you think you can. I would never have been able to do that myself.” – Participant, Study 17 |
| 15 | Participants saw providers as critical sources of support throughout their cancer journey, transcending the role of an ‘exercise provider.’ Providers' roles included acting as conduits between participants and the healthcare system, explaining complex medical concepts in accessible ways, answering questions, and simply caring and listening. Many participants recalled generating close relationships with their providers, supporting exercise behaviour change. | “She held my hand all the way through it. She made me feel as though I was the only one that was important. If I had a question about anything she’d sort it, and I could sit face-to-face with her and she had time. She was just perfect for me…She was a good listener, she took everything I said seriously.” – Participant, Study 2. “The lung nurse would sometimes come in with me, so that if I had a question afterwards I could ring them up. And say…what did that mean? …The lung nurse helpline for me was absolutely amazing” – Participant, Study 2 |
| Theme 3: The value of exercise programs | | |
| *Taking an active role in one’s own healthcare* | | |
| 16 | For many participants, a diagnosis of lung cancer was a ‘call to action’ to take control of their health through exercise. Participating in exercise programs facilitated many participants' generation of an internal locus of control and taking an active role in their healthcare. For some participants, participating helped them remain positive and hopeful, provided an opportunity to distance or distract themselves from a lung cancer diagnosis, and supported a shift in mindset towards wellness and recovery rather than sickness. | “It’s an addition to you health and life. It makes rehabilitation a little easier and more challenging. It focuses more on recovery than on your problems.” – Participant, Study 5. “You feel more co-responsible. Well then it’s nice that you can show you are doing well and that you try your best.” – Participant., Study 5. “It’s all very frightening and scary. What is going to happen? Will I wake up after the operation? Can I still do the same as I did? There is a lot going through you head and it’s pretty scary, to be honest. At that moment, health professionals and such a motion sensor around you ankle helps enormously. You get feedback and it gives you something to hold on to.” – Participant, Study 5 |
| 17 | Program components such as exercise prescription/advice, activity monitoring tools, and symptom self-management education facilitated habit and skill-building, the development of exercise self-efficacy, and a ‘toolbox’ that supported participants to exercise independently. For many participants, this translated into plans to remain physically active after program discharge. | “…Others described additional confidence gained from the daily diary because it allowed participants the opportunity to visually see and “track” their progress with tools provided in the study, such as the pulse oximeter.” – Author interpretation. “This program was well worth it! It helped both my wife and me to start and continue a regular daily exercise program… We plan to continue our exercises as we go through chemo and beyond.” – Participant, Study 12 |
| *Social connectivity* | | |
| 18 | Lung cancer group programs facilitated important opportunities for participants to forge friendships, a sense of belonging, and camaraderie through shared experiences of a life-altering diagnosis. Participants reported specific benefits of group-based training, such as improvements to social wellbeing, contextualising their experiences, learning from others’ experiences, building hope, and a sense of freedom to discuss and share grief. In some instances, participants became critical sources of social support for each other | “I really appreciated the exercise class, also socially. After cancer, you feel very vulnerable, but I felt comfortable in the group. It was good that we had all been through the same thing – the same illness and the same surgery – because we could compare notes and find out from each other how we were coping with various problems. That way we were a good support for each other.” – Participant, Study 18 |
| *Enjoyment* | | |
| 19 | Many participants found enjoyment in exercise participation. | “We all enjoy, and I’m not watching and looking at the clock like oh, I need to go home, no, I’m never do that, I enjoy 100% doing the exercise.” – Participant, Study 7 |
| *Benefits to physical and mental health* | | |
| 20 | Most participants felt that participation directly improved their motivation to be physically active, and/or their physical activity levels. | “From the start of the programme, I could feel more positive about myself. Also, it encouraged me to do exercises at home which I probably would not have done.” – Participant, Study 2 |
| 21 | The benefits of participating in exercise programs were seen to ‘go beyond’ improvements to physical health, with many participants, providers and caregivers recalling the multifaceted healing capacity of exercise. Many participants voiced benefits to their physical and/or mental health, which were seen as interconnected. Successfully completing an exercise session often contributed to a sense of instantly feeling ‘better.’ Physical benefits included reduced symptoms such as breathlessness, pain and fatigue; improved symptom self-management, physical function, functional capacity, fitness, muscle strength and/or tone, and flexibility; and prevention of deterioration/functional maintenance. Mental benefits included improved self-esteem, self-efficacy, coping skills, relaxation, cognition, positivity, emotional regulation, and reduced stress and anxiety. | “First of all, I now have more clout. I think that I feel better and I don't feel as worthless, actually. Some of my back pain has disappeared. I used to take 8 painkillers a day for my back but I don't do that anymore. The program helped with my physical strength; I gained 5 kilos since I started the program.” - Participant, Study 1  “For the first time in my (adult) life I have not needed my walker. . .I can get around 90% of the day without a walker.” – Participant, Study 12  “What if I just stay at home, keep thinking about my disease, and counting the days until I die? You cannot live in this manner. Once you go outside, talk with others, or perform walking exercise, your mood gets better.” - Participant, Study 13 |
| 22 | Many participants generated increased exercise self-efficacy through repeated exercise practice and supported exercise skill-building. | “Having come through the first month, which is the most difficult period after surgery, all participants at the interview expressed more confidence in their exercise ability and stated that they had an exercise plan for the future.” – Author interpretation, Study 13 |
| 23 | All stakeholder groups reported that caregivers benefitted broadly from participating and/or supporting their loved ones, including increased motivation to be physically active themselves, improved physical and/or mental well-being, and an opportunity to support and/or re-build relationships with their loved one. They also reported improved knowledge, such as an increased understanding of their loved ones’ health and diagnosis, and of ways to support and care for their loved ones. | “One family member with serious chronic illnesses, a son to a female survivor, joined because ‘he figured it would help her.’” – Author interpretation. He added, “But since I came to the group, I found that it helped me as well.” – Family member participant. “[My husband] got more insight into my condition when he could see that others had the same issues, and he became more familiar with how to approach these symptoms.” – Participant, Study 16 |
| 24 | Some participants of pre- and rehabilitation programs believed that exercise was a significant driver in their surgical readiness and improving and/or hastening their postoperative recovery. | “If it were up to me, I would love to come every day, and for more hours.” – Participant, Study 1  “Participant Informed Future Directions: Need for an ongoing / longer duration program.” – Author interpretation, Study 20 |
| 25 | Most, but not all, participants felt that they benefited from participating in programs. Providers also believed that programs were beneficial. Some participants reported no perceived benefits, and others felt they would have been active despite their enrolment in a program and, therefore, that the program was unnecessary for them. | “When asked how they felt now compared to the start of the program, participants had varied responses from feeling the same, to feeling more calm and comforted, to feeling better in many areas.” – Author interpretation, Study 3 |
| Theme 4: Facilitating exercise behaviour change | | |
| *Key enablers of exercise* | | |
| 26 | Generating exercise habits and finding ways to integrate exercise into the daily routine were key enablers of lasting behaviour change. Program structure and a requirement to attend scheduled sessions were key extrinsic motivating factors for participation for some participants, whereas for others, the flexibility home-based programs offered to choose when to exercise was an enabler. | “Home also enabled a more flexible routine, allowing exercise to be balanced with rest or arranged around how they were feeling.” – Author interpretation, Study 6  “The supervised training had scheduled hours and participation was mandatory as required in other team sports. Furthermore, the participants were aware that the other participants expected them to show up for training, unlike comparative training forms in fitness centres.” – Author interpretation, Study 1 |
| 27 | Program providers were seen as key enablers of participation. Many participants felt accountable or committed to their providers, facilitating adherence. Participants of centre-based programs reported relying on external supervision to sustain motivation. Conversely, for participants of home-based programs, regular remote reviews with providers, such as phone calls, served a similar purpose. Across all exercise settings, participants valued receiving feedback on progress and activity levels from providers. Education and reassurance from expert providers regarding the importance and safety of exercise were critical enablers of exercise uptake and adherence. | “I knew the coach would be calling then I could tell her how much more I had done and track my progress.” – Participant, Study 22. “I’m someone who if I commit to something, I feel compelled to do it, and having the check-in meant that, I’m an honest person, but there’s someone monitoring me on top of that.” – Participant, Study 22. “The phone call that you would get allowed you to talk about the plan that you had set. It helped me stay focused because I knew that I had somebody to answer to.” - Participant, Study 22 |
| 28 | Clear activity goals (e.g., daily step targets) can drive behaviour change, and many participants saw activity monitoring tools such as pedometers as critical sources of extrinsic motivation. These tools provided a visual tool, enabled the self-monitoring of activity levels and recovery, and served as proof of ability. Achieving and/or exceeding exercise goals was an important driver of self-efficacy and a sense of achievement. | “…but the more I do, the more I have enjoyed doing… up in the morning, have my shower, put my clothes on, put my pedometer, just became second nature, it’s like if I don’t have it on, I feel a little bit lost, it’s like my little buddy, my little buddy goes everywhere with me you know and I just you know, and I’m always checking it just to make sure.” – Participant, Study 4 |
| 29 | Positive outcome expectancy was key to motivation and hope among many participants. Participants and providers tended to report a shared understanding of the importance and efficacy of exercise for patients with lung cancer and other medical conditions. Believing that the program would positively influence health, well-being or longevity, and/or prevent deterioration/decline, was a strong enabler of program sign-up and adherence, and experiencing perceived benefits of participation was additionally a strong enabler of adherence/sustained exercise uptake. | The patients’ motivation for participation included their expectations of physical and social benefits and the security of having professionals present: “When you’re missing a piece of your lung, it’s sensible to take advantage of any remaining lung capacity” – Participant, Study 17 |
| 30 | Many participants saw having pre-existing exercise habits before their lung cancer diagnosis as a key enabler to recommencing and sustaining activity, exercise self-efficacy, and increased exercise intensity, particularly when facing barriers. | “I enjoyed, I enjoy walking so that was a big part of the, the program so, um, you know, that that was, um, something that I enjoyed to do anyway, it wasn’t a big chore to have to do it…” – Participant, Study 6 |
| 31 | Optimising symptom control and support to monitor and self-manage symptoms in collaboration with providers and other healthcare professionals enabled exercise uptake and adherence. Some participants displayed an inherent persistence and self-efficacy to exercise despite severe symptoms and side effects, while others generated this through the support of their providers. | “Patients and their family members trusted and relied on the information provided by the nurse who motivated and supported them... The nurse's follow-up calls were considered essential by the participants because so many unexpected problems arose that made them worried and afraid. These problems often resulted in exercise termination until the nurse answered their questions, explained that some discomfort was normal for postsurgical patients, and told the participants how to manage these issues.” – Author interpretation, Study 13 |
| 32 | Encouragement and support from loved ones increased motivation for some participants. Participating in exercise alongside loved ones also increased motivation and enjoyment. | “…I enjoy walking so that was a big part of the, the program so, um, you know, that that was, um, something that I enjoyed to do anyway, it wasn’t a big chore to have to do it…” – Participant, Study 6 |
| *Key barriers to exercise* | | |
| 33 | Symptoms such as fatigue, pain, and breathlessness were commonly experienced barriers to exercising. Adjuvant therapy side effects similarly often prevented participants from exercising, and participants recalled the ‘destructive’ nature of adjuvant therapies (especially chemotherapy) to both their bodies and their daily routines. Side effects associated with exercising such as exacerbating symptoms or inducing muscle pain/fatigue, and comorbidities, also influenced participation. The impacts of these barriers varied, sometimes forcing exercise regression or modification and/or completely inhibiting participation. | “Many of the participants faced adverse symptoms as a consequence of their cancer and its treatment, such as fatigue and nausea. These symptoms compromised their ability to adhere to their personal goals and often led to a requirement to adjust targets and revise expectations for subsequent weeks.” – Author interpretation. “The chemotherapy … wrecks everything… I feel rotten, I just want to go in to my bed, pull the clothes over me.” – Participant, Study 19 |
| 34 | Time was a barrier to exercising. The unpredictability of cancer treatment pathways, particularly planned and unplanned medical appointments, influenced participants’ ability to participate in both home and centre-based programs. Additionally, exercise was often deprioritised in favour of other competing priorities, such as employment, caregiving, and socialising. | “The need to schedule and attend multiple [medical] appointments in the preoperative setting made it difficult to set aside time for walking. Unexpected life events, such as car accidents, additional medical issues, and unanticipated long-distance travels limited participants' ability to engage in the intervention.” – Author interpretation, Study 23 |
| 35 | Poor weather (e.g., rain, wind and extreme heat and/or cold) inhibited many participants' ability and/or willingness to participate in home-based exercise. | “It was a challenge for patients to plan and perform the exercise program at home and to go for walks when it was dark outside, and the weather was bad.” – Author interpretation, Study 15 |
| 36 | For participants of one study, social and cultural beliefs about emphasising rest in recovery were a barrier to postoperative exercise. | “Two participants described their family members as barriers; they did not attend exercise education and had negative attitudes toward the exercise. In addition, they firmly believed that patients should rest in bed after surgery. To make matters worse, the participants lacked self-efficacy and tended to be obedient toward family members.” – Author interpretation, Study 13. “Is it necessary to do such high amount and intensity of exercise after surgery? It is possible that the intervention is too ambitious. In the traditional Chinese view, it would be better that a person has a good rest after surgery.” – Participant, Study 13 |
| 37 | Participants recalled difficulty generating the intrinsic motivation required to ‘get moving’, often citing their own ‘laziness’ or lack of discipline as a barrier. Low exercise self-efficacy similarly prevented some participants from exercising. Participants of centre-based programs especially often reported challenges initiating independent exercise outside of their supervised sessions due to lacking motivation and/or confidence. | “It is always easier to do that sort of thing under supervision. We’re all a bit slack when we’re not being boot-camped around by somebody else. And obviously, it may be in conjunction with other people undergoing a similar exercise regime.” – Participant, Study 2. “I’m not disciplined enough… I need someone there.” – Participant, Study 2 |
| 38 | Pre-existing habits relating to exercise influenced participation. Participants who reported having a relatively sedentary lifestyle before commencing their program felt this was a barrier to exercise uptake. In contrast, some participants who viewed themselves as active felt that an exercise program would not necessarily benefit them. | “A lack of personal history of physical activity engagement was a common intrapersonal factor that resulted in disinterest and dislike of walking. For some patients and [caregivers], preference for sedentary lifestyles, coupled with lack of socialization, posed a significant challenge to physical activity engagement. Others preferred leisurely activities over a more structured exercise. Because they were “active” leisurely, the need for structured walking programs were not necessary.” – Author interpretation, Study 23 |
| 39 | Intervention acceptability was a barrier to exercise participation, including participants’ lack of enjoyment of exercise, preference to remain sedentary, and/or disinterest in the type of exercise prescribed. A minority of participants felt that they did not require such a program, and/or that it would not help them. Some participants and providers also questioned intervention appropriateness at times, for example, the appropriateness of prescribing high-intensity exercise to participants early after surgery or those with short life expectancies. | “Some patients declined to participate in the exercise program because they did not want to use all their remaining time on transportation to the exercise facility. Physiotherapists reported ethical concerns when asking patients to exercise vigorously in the last period of their lives.” – Author interpretation, Study 15 |
| 40 | Participants and providers encountered several logistical barriers, often specific to intervention design. Participants of centre-based programs were limited by travel and parking requirements, with some living considerable distances away. Prehabilitation programs were limited by the time between diagnosis and surgery. | “I…had a thing off the [program] nurses…saying about did I want to go there for exercise and things. And I thought I’m going in hospital for an operation soon. It wouldn’t, didn’t apply to me at the time, no good going to them because I was going in hospital within a week or two.” – Participant, Study 2 |
| 41 | Participants of programs incorporating a technological element, such as online symptom monitoring or activity tracking technologies, encountered specific barriers, such as a lack of digital skills and/or self-efficacy, discomfort, and a general dislike of the prescribed technology. | There was a long, idiosyncratic list of things people did not like or found “confusing about the fitness device. Twenty-two participants (79%) reported at least one aspect of the fitness device that they disliked, and eight participants (29%) reported at least one thing they did not understand regarding the device.” – Author interpretation, Study 8 |

**Table S17: Evidence Profile Table**

| # | Summarised review finding | Methodological limitations | Coherence | Adequacy | Relevance | GRADE-CERQual assessment of confidence | Supporting studies |
| --- | --- | --- | --- | --- | --- | --- | --- |
| Theme 1: Components of exercise program design | | | | | | | |
| *Exercise prescription* | | | | | | | |
| 1 | Programs that prescribed exercise at an ‘achievable’ intensity and provided ‘controlled exposure’ to exercise supported participants in building exercise self-efficacy. Participants also appreciated provider-led exercise progression throughout the program. | Minor concerns | Minor concerns | Moderate concerns | No/Very minor concerns | Moderate confidence | (4) (6) (7) (11) (16) |
|  |  | **Explanation:** Minor concerns regarding methodological limitations because all studies raised concerns regarding reflexivity. Researcher role was not seen to influence this stated preference, and so the overall concern was rated as minor. 1 study raised concerns regarding methodology appropriateness and data analysis reporting/rigour, although it did not contribute heavily to the finding. | **Explanation:** Minor concerns regarding coherence because the finding is broadly supported and mostly descriptive. In 2 studies, appreciation regarding provider-led progression is either implied or unclear. Some included studies did not discuss the correlation between an achievable intensity and increased self-efficacy in-depth. | **Explanation:** Moderate concerns regarding adequacy because only 5 studies contributed to the finding, and 1 study only contributed in part. Given that the finding is mainly descriptive, the level of concern was rated as minor. | **Explanation:** No/very minor concerns regarding relevance because the contextual factors were relevant to those specified in the review question. | **Explanation:** Due to minor concerns regarding methodological limitations and coherence, moderate concerns regarding adequacy, and no/very minor concerns regarding relevance. |  |
| 2 | Participants valued individualisation and tailoring, including individualised exercise prescription, goal setting, and education. Individualisation was seen as an enabler of exercise participation, and some participants gleaned additional motivation to participate in exercise by feeling individually cared for. | Minor concerns | No/Very minor concerns | No/Very minor concerns | No/Very minor concerns | High confidence | (2) (4) (6) (7) (10) (19) (20) |
|  |  | **Explanation:** Minor concerns regarding methodological limitations because all studies raised concerns regarding reflexivity, and 2 regarding the rigour and/or reporting of data analysis. | **Explanation:** No/very minor concerns regarding coherence because the finding is well supported by and reflects the variation of the data. | **Explanation:** No/very minor concerns regarding adequacy because while the supporting data was often relatively superficial, the finding was simple and descriptive. | **Explanation:** No/very minor concerns regarding relevance because 3 studies did not specify the program provider; however, all other contextual factors were relevant to those specified in the review question. | **Explanation:** Due to minor concerns regarding methodological limitations, and no/very minor concerns regarding coherence, adequacy, and relevance. |  |
| 3 | Walking was an accessible, simple and enjoyable form of exercise highly valued by participants. | Minor concerns | No/Very minor concerns | Moderate concerns | No/Very minor concerns | Moderate confidence  **Explanation:** Due to minor concerns regarding methodological limitations, no/very minor concerns regarding coherence and relevance, and moderate concerns regarding adequacy. | (4) (6) (13) |
|  |  | **Explanation:** Minor concerns regarding methodological limitations because of concerns about reflexivity across all studies, which may have influenced participants' stated exercise preferences. | **Explanation:** No/very minor concerns regarding coherence because the descriptive finding was broadly supported by all contributing studies. Data is varied and describes all 3 components of the finding. | **Explanation:** Moderate concerns regarding adequacy because only 3 studies contributed to this finding, although 2 provided rich data. | **Explanation:** No/very minor concerns regarding relevance because the contextual factors were relevant to those specified in the review question. |  |  |
| 4 | Preferences regarding exercise types were variable, highlighting the importance of autonomy and providing various exercise options to allow for individual preferences. Variety was seen as an enabler of sustained exercise enjoyment and behaviour change, and some participants viewed programs that did not offer this flexibility as regimented and boring. | Moderate concerns  **Explanation:** Moderate concerns regarding methodological limitations because all studies raised concerns regarding reflexivity, 3 regarding data analysis reporting and/or rigour, and 1 regarding recruitment transparency. | Minor concerns  **Explanation:** Minor concerns regarding coherence because the variation in preference is reflected in the finding; however, boredom is only expressed specifically in 1 study. | No/Very minor concerns  **Explanation:** No/very minor concerns regarding adequacy because 8 studies contributed to this finding, with appropriate richness to support the explanatory nature of the finding. | No/Very minor concerns  **Explanation:** No/very minor concerns regarding relevance because 1 study did not specify the program provider; however, all other contextual factors were relevant to those specified in the review question. | High confidence  **Explanation:** Due to moderate concerns regarding methodological limitations, minor concerns regarding coherence, and no/very minor concerns regarding adequacy and relevance. | (4) (7) (8) (12) (14) (19) (20) (22) |
| 5 | Some participants and providers of pre- and rehabilitation programs desired a longer program and/or more frequent sessions because they believed that longer programs were more beneficial. | Moderate concerns  **Explanation:** Moderate concerns regarding methodological limitations because all studies raised concerns regarding reflexivity, 2 regarding data analysis reporting and/or rigour, and 1 regarding data collection. | Moderate concerns  **Explanation:** Moderate concerns regarding coherence because outliers (e.g., participants who were satisfied with their program length/duration) were not described in detail in the finding. The finding is, however, broadly supported by all contributing studies. | Moderate concerns  **Explanation:** Moderate concerns regarding adequacy because 4/5 studies contribute relatively superficial data; however, the finding is descriptive in nature. | No/Very minor concerns  **Explanation:** No/very minor concerns regarding relevance because the contextual factors were relevant to those specified in the review question. | Moderate confidence  **Explanation:** Due to moderate concerns regarding methodological limitations, coherence and adequacy, and no/very minor concerns regarding relevance. | (1) (4) (16) (19) (20) |
| *Exercise setting* | | | | | | | |
| 6 | Participants in group programs saw them as valuable and an essential source of motivation, socialisation, and support. One study discussed group size and reported that participants tended to prefer smaller group sizes to foster familiarity, intimacy, and closeness and reduce feelings of intimidation and overwhelm. Participants valued face-to-face supervision and opportunities for one-on-one support from program providers to ensure they completed prescribed exercises correctly. Some participants of centre-based programs/ desired an individualised home exercise program to support behaviour change and independent exercise outside supervised sessions. | Serious concerns  **Explanation:** Serious concerns regarding methodological limitations because all studies had raised concerns regarding reflexivity, 2 regarding methodology appropriateness, 2 regarding data collection, and 4 regarding data analysis. | No/Very minor concerns  **Explanation:** No/very minor concerns regarding coherence because the finding is broadly supported by the data and reflects data variation. One participant (Crandall 2016) reportedly found exercising in a larger group overwhelming and subsequently withdrew, which cannot necessarily be interpreted as a preference for a smaller group setting and may reflect an outlier. | No/Very minor concerns  **Explanation:** No/very minor concerns regarding adequacy because while only 7 studies contributed to the finding, the supporting data is rich, and the finding is descriptive in nature. | No/Very minor concerns  **Explanation:** No/very minor concerns regarding relevance because all contextual factors align with the research question. | Moderate confidence  **Explanation:** Due to serious concerns regarding methodological limitations, and no/very minor concerns regarding coherence, adequacy, and relevance. | (1) (3) (4) (7) (16) (18) (20) |
| 7 | Participants of home-based programs saw them as valuable. Many, especially participants with more advanced lung cancer, viewed home-based exercise as less burdensome than attending centre-based programs. | Moderate concerns  **Explanation:** Moderate concerns regarding methodological limitations because 2/3 studies raised concerns regarding the rigour and/or reporting of data analysis, 1 regarding recruitment transparency, and all 3 raised minor concerns regarding reflexivity. | Minor concerns  **Explanation:** Minor concerns regarding coherence because some ambiguous data is not discussed in the finding; however, the data largely supports the descriptive finding. | Moderate concerns  **Explanation:** Moderate concerns regarding adequacy because only 3 studies were included, with 2 contributing relatively thin data. | Moderate concerns  **Explanation:** Moderate concerns regarding relevance because 2/3 programs were offered in mixed settings (e.g., home and centre-based), and 2/3 were conducted in Denmark. | Moderate confidence  **Explanation:** Due to moderate concerns regarding methodological limitations, adequacy, and relevance, and minor concerns regarding coherence. | (1) (6) (15) |
| 8 | Participants of digital programs had mixed experiences, with some reporting drawbacks such as inadequate tailoring, flexibility, feedback, and support. | Serious concerns  **Explanation:** Serious concerns regarding methodological limitations because no studies discussed researcher reflexivity, 3/4 raised concerns regarding recruitment transparency, and 3/4 regarding data analysis reporting and/or rigor. | Minor concerns  **Explanation:** Minor concerns regarding coherence because the supporting data mostly supports the limitations of digital programs. Data was varied, and the four broad drawbacks of digital programs were supported. | Moderate concerns  **Explanation:** Moderate concerns regarding adequacy because only 4 studies contributed, with 2/4 providing relatively superficial data. | Minor concerns  **Explanation:** Minor concerns regarding relevance because 2/4 studies did not specify a program provider, 3/4 were conducted in the Netherlands, and 4/4 were conducted in Europe. | Low confidence  **Explanation:** Due to serious concerns regarding methodological limitations, minor concerns regarding coherence and relevance, and moderate concerns regarding adequacy. | (5) (9) (10) (24) |
| *Outcome measures* | | | | | | | |
| 9 | Participants viewed participating in outcome measurement assessments as a way to improve motivation, resilience and self-efficacy by allowing participants to exceed their own expectations, demonstrating what is possible, and providing an objective measurement of their capacity and progress. | Moderate concerns  **Explanation:** Moderate concerns regarding methodological limitations because 2/4 studies raised concerns regarding data analysis reporting and/or rigour, 1 regarding recruitment transparency, and all raised concerns regarding reflexivity. | No/Very minor concerns  **Explanation:** No/very minor concerns regarding coherence because the finding is reflective of the data and variation. | Minor concerns  **Explanation:** There are minor concerns regarding adequacy because although only 4 studies were included, the finding is descriptive in nature, and the supporting data is rich. | No/Very minor concerns  **Explanation:** No/very minor concerns regarding relevance because the contextual factors were relevant to those specified in the review question. | High confidence  **Explanation:** Due to moderate concerns regarding methodological limitations, no/very minor concerns regarding coherence and relevance, and minor concerns regarding adequacy. | (4) (7) (15) (19) |
| 10 | Some participants desired provider feedback regarding their performance in outcome measurements and progress throughout the program. | Moderate concerns  **Explanation:** Moderate concerns regarding methodological limitations because 2/3 studies raised concerns regarding data analysis reporting and/or rigour, all raised concerns regarding reflexivity, and 1 raised concerns regarding recruitment transparency and data collection. | No/Very minor concerns  **Explanation:** No/very minor concerns regarding coherence because the finding is broadly supported by the data. | Moderate concerns  **Explanation:** Moderate concerns regarding adequacy because 3 studies provided superficial data. As the review finding is simple and descriptive, the level of concern was rated as moderate. | No/Very minor concerns  **Explanation:** No/very minor concerns regarding relevance because while 2/3 studies did not specify an program provider, all other contextual factors were relevant to those specified in the review question. | Moderate confidence  **Explanation:** Due to moderate concerns regarding methodological limitations and adequacy, and no/very minor concerns regarding coherence and relevance. | (6) (9) (20) |
| 11 | Participants' views regarding the acceptability and utility of specific outcome measures were varied. In one study, participants and providers viewed physical outcome measures such as the Six-Minute Walk Test and the 60-second Sit-to-Stand Test as useful, relevant tools to monitor fitness and progress. Providers in another study reported that the Ekblom-Bak test was too burdensome for participants to complete. Participants of two studies found Patient Reported Outcomes (PROs) overly burdensome, cumbersome, and/or difficult to complete, whereas participants of another found them acceptable. | Serious concerns  **Explanation:** Serious concerns regarding methodological limitations because all studies raised concerns regarding researcher reflexivity, and 2 raised concerns regarding recruitment transparency, 1 regarding data collection, and 2 regarding data analysis. | Minor concerns  **Explanation:** Minor concerns regarding coherence because 3 of the studies did not explore/report what participants meant in detail when they expressed the outcome measures were unliked/burdensome. However, the finding reflects the varied data and describes ambiguities or outliers. | Moderate concerns  **Explanation:** Moderate concerns regarding adequacy because only 4 studies contributed to the finding and 3 of these studies contributed thin data. Because the finding is descriptive in nature, the level of concern was judged as moderate. | No/Very minor concerns  **Explanation:** No/very minor concerns regarding relevance because while 1 study did not specify the program provider, all other contextual factors were relevant to those specified in the review question. | Low confidence  **Explanation:** Due to serious concerns regarding methodological limitations, minor concerns regarding coherence, moderate concerns regarding adequacy, and no/very minor concerns regarding relevance. | (9) (12) (15) (19) |
| *Program timing* | | | | | | | |
| 12 | Participants of some prehabilitation-only programs desired program continuation and support postoperatively to guide them to resume exercise and normal activities due to postoperative symptoms and physical inactivity. | No/Very minor concerns  **Explanation:** No/very minor concerns regarding methodological limitations because neither adequately addressed reflexivity. Researcher role was judged as unlikely to influence this stated preference, and so the overall concern was rated as very minor. | No/Very minor concerns  **Explanation:** No/very minor concerns regarding coherence because the finding broadly represents the data and its variation. | Moderate concerns  **Explanation:** Moderate concerns regarding adequacy because only 2 studies contributed to this finding. | No/Very minor concerns  **Explanation:** No/very minor concerns regarding relevance because the contextual factors were relevant to those specified in the review question. | Moderate confidence  **Explanation:** Due to no/very minor concerns regarding methodological limitations, coherence, and relevance, and moderate concerns regarding adequacy. | (4) (21) |
| Theme 2: Providers of exercise programs | | | | | | | |
| *Expertise* | | | | | | | |
| 13 | Participants valued program providers’ expertise independent of their specialty/discipline, including expert education, guidance, monitoring, and exercise progression, regression, and modification. Support from a qualified expert improved confidence, safety, and trust. | Moderate concerns  **Explanation:** Moderate concerns regarding methodological limitations because of concerns regarding reflexivity across all studies. 4 studies raised additional concerns regarding data analysis and 1 regarding potential recruitment transparency. | No/Very minor concerns  **Explanation:** No/very minor concerns regarding coherence because the finding reflects the complexity and variation of, and is well supported by, the data. | No/Very minor concerns  **Explanation:** No/very minor concerns regarding adequacy because 12 studies with overall rich data contributed. | No/Very minor concerns  **Explanation:** No/very minor concerns regarding relevance because while some studies did not specify an program provider, all other contextual factors were relevant to those specified in the review question. | High confidence  **Explanation:** Due to moderate concerns regarding methodological limitations, and no/very minor concerns regarding coherence, adequacy, and relevance. | (4) (6) (7) (10) (12) (13) (15) (17) (19) (20) (21) (22) |
| *Role* | | | | | | | |
| 14 | Providers played a key role in supporting, encouraging, and educating participants to exercise and were seen as key instigators of health behaviour change by acting as important sources of extrinsic motivation and supporting participants to build exercise self-efficacy. | Moderate concerns  **Explanation:** Moderate concerns regarding methodological limitations because of concerns regarding researcher reflexivity in all studies; 2 studies raised concerns regarding data analysis reporting and/or rigour, and 1 regarding recruitment transparency. | No/Very minor concerns  **Explanation:** No/very minor concerns regarding coherence because the finding reflects the complexity and variation of the data. | No/Very minor concerns  **Explanation:** No/very minor concerns regarding adequacy because 9 studies contributed to this finding. The finding is explanatory in nature and supported by rich data. | No/Very minor concerns  **Explanation:** No/very minor concerns regarding relevance because while 1 study did not specify the program provider, all other contextual factors were relevant to those specified in the review question. | High confidence  **Explanation:** Due to moderate concerns regarding methodological limitations, and no/very minor concerns regarding coherence, adequacy, and relevance. | (2) (6) (12) (13) (15) (17) (19) (21) (22) |
| 15 | Participants saw providers as critical sources of support throughout their cancer journey, transcending the role of an ‘exercise provider.’ Providers' roles included acting as conduits between participants and the healthcare system, explaining complex medical concepts in accessible ways, answering questions, and simply caring and listening. Many participants recalled generating close relationships with their providers, supporting exercise behaviour change. | Minor concerns  **Explanation:** Minor concerns regarding methodological limitations because of concerns related to researcher reflexivity. 1 study (Langballe 2023) raised additional concerns relating to recruitment and data analysis; however, because this study did not contribute new findings, the level of concern judged to be minor. | No/Very minor concerns  **Explanation:** No/very minor concerns regarding coherence because the finding is well supported by the data and reflects its variation. | No/Very minor concerns  **Explanation:** No/very minor concerns regarding adequacy because while only 7 studies contributed, the data was rich and supported the explanatory nature of the finding. | No/Very minor concerns  **Explanation:** No/very minor concerns regarding relevance because while 1 study did not specify the program provider, all other contextual factors were relevant to those specified in the review question. | High confidence  **Explanation:** Due to minor concerns regarding methodological limitations and no/very minor concerns regarding coherence, adequacy, and relevance. | (2) (6) (11) (12) (13) (15) (22) |
| Theme 3: The value of exercise programs | | | | | | | |
| *Taking an active role in one’s own healthcare* | | | | | | | |
| 16 | For many participants, a diagnosis of lung cancer was a ‘call to action’ to take control of their health through exercise. Participating in exercise programs facilitated many participants' generation of an internal locus of control and taking an active role in their healthcare. For some participants, participating helped them remain positive and hopeful, provided an opportunity to distance or distract themselves from a lung cancer diagnosis, and supported a shift in mindset towards wellness and recovery rather than sickness. | Moderate concerns  **Explanation:** Moderate concerns regarding methodological limitations because of concerns raised regarding researcher reflexivity across all studies, data analysis rigour and/or reporting in 3 studies, and recruitment transparency in 1 study. | No/Very minor concerns  **Explanation:** No/very minor concerns regarding coherence because the finding reflects the complexity and variation of the data. | No/Very minor concerns  **Explanation:** No/very minor concerns regarding adequacy because 12 studies contributed rich data to support this explanatory finding. | No/Very minor concerns  **Explanation:** No/very minor concerns regarding relevance because the contextual factors were relevant to those specified in the review question. | High confidence  **Explanation:** Due to moderate concerns regarding methodological limitations and no/very minor concerns regarding coherence, adequacy, and relevance. | (1) (4) (5) (6) (7) (12) (11) (16) (18) (19) (21) (22) |
| 17 | Program components such as exercise prescription/advice, activity monitoring tools, and symptom self-management education facilitated habit and skill-building, the development of exercise self-efficacy, and a ‘toolbox’ that supported participants to exercise independently. For many participants, this translated into plans to remain physically active after program discharge. | Moderate concerns  **Explanation:** Moderate concerns regarding methodological limitations because of concerns raised regarding researcher reflexivity across all supporting studies and data analysis reporting and/or rigour in 2 studies. | Minor concerns  **Explanation:** Minor concerns regarding coherence because the finding broadly reflects the variation of the data. However, goal setting was discussed in three studies, which was not specifically highlighted in this finding. | Minor concerns  **Explanation:** Minor concerns regarding adequacy because the finding is supported by 9 studies, 8 of which contributed rich data. | No/Very minor concerns  **Explanation:** No/very minor concerns regarding relevance because while 1 study did not specify an program provider, all other contextual factors were relevant to those specified in the review question. | Moderate confidence  **Explanation:** Due to moderate concerns regarding methodological limitations, minor concerns regarding coherence and adequacy, and no/very minor concerns regarding relevance. | (2) (4) (11) (12) (13) (16) (17) (19) (20) |
| *Social connectivity* | | | | | | | |
| 18 | Lung cancer group programs facilitated important opportunities for participants to forge friendships, a sense of belonging, and camaraderie through shared experiences of a life-altering diagnosis. Participants reported specific benefits of group-based training, such as improvements to social wellbeing, contextualising their experiences, learning from others’ experiences, building hope, and a sense of freedom to discuss and share grief. In some instances, participants became critical sources of social support for each other. | Serious concerns  **Explanation:** Serious concerns regarding methodological limitations because of concerns raised regarding researcher reflexivity in all studies and concerns regarding the appropriateness of the methodology (2 studies), data analysis (5 studies), and data collection (2 studies) methods. | No/Very minor concerns  **Explanation:** No/very minor concerns regarding coherence because the finding reflects the complexity and variation of the data. | Minor concerns  **Explanation:** Minor concerns regarding adequacy because 9 supporting studies provided mostly rich data. | No/Very minor concerns  **Explanation:** No/very minor concerns regarding relevance because while 2 studies did not specify an program provider, all other contextual factors were relevant to those specified in the review question. | Moderate confidence  **Explanation:** Due to serious concerns regarding methodological limitations, minor concerns regarding adequacy, and no/very minor concerns regarding coherence and relevance. | (1) (2) (3) (4) (7) (16) (17) (18) (20) |
| *Enjoyment* | | | | | | | |
| 19 | Many participants found enjoyment in exercise participation. | Minor concerns  **Explanation:** Minor concerns regarding methodological limitations because of minor concerns relating to researcher reflexivity across all studies, which may have influenced participants’ likelihood to discuss program enjoyment. 1 study also raised concerns regarding methodology appropriateness and data analysis. | Minor concerns  **Explanation:** Minor concerns regarding coherence because whilst the data is varied and broadly supports the descriptive finding, enjoyment was often not described in great detail. | Moderate concerns  **Explanation:** Moderate concerns regarding adequacy because only 5 studies supported the finding, and 3 contributed relatively thin data. Given the descriptive nature of the finding, the concern level was judged to be moderate. | No/Very minor concerns  **Explanation:** No/very minor concerns regarding relevance because the contextual factors were relevant to those specified in the review question. | Moderate confidence  **Explanation:** Due to minor concerns regarding methodological limitations and coherence, moderate concerns regarding adequacy, and no/very minor concerns regarding relevance. | (4) (6) (7) (13) (19) |
| *Benefits to physical and mental health* | | | | | | | |
| 20 | Most participants felt that participation directly improved their motivation to be physically active, and/or their physical activity levels. | Serious concerns  **Explanation:** Serious concerns regarding methodological limitations because of minor concerns raised regarding researcher reflexivity across all studies, which may have influenced participants' recounts of program efficacy. 7 studies raised additional concerns regarding data analysis reporting and/or rigour and 3 regarding recruitment transparency. | No/Very minor concerns  **Explanation:** No/very minor concerns regarding coherence because the descriptive finding broadly captures the supporting data. | No/Very minor concerns  **Explanation:** No/very minor concerns regarding adequacy because whilst some of the supporting data is superficial and thin, 13 studies support the finding. Given the descriptive nature of the finding, the level of concern was judged as very minor. | No/Very minor concerns  **Explanation:** No/very minor concerns regarding relevance because while 2 studies did not specify an program provider, all other contextual factors aligned. There was a good variation of exercise program delivery modes/types to support the broad finding. | Moderate confidence  **Explanation:** Due to serious concerns regarding methodological limitations, and no/very minor concerns regarding coherence, adequacy, and relevance. | (1) (2) (4) (5) (6) (7) (12) (13) (14) (15) (17) (19) (20) |
| 21 | The benefits of participating in exercise programs were seen to ‘go beyond’ improvements to physical health, with many participants, providers and caregivers recalling the multifaceted healing capacity of exercise. Many participants voiced benefits to their physical and/or mental health, which were seen as interconnected. Successfully completing an exercise session often contributed to a sense of instantly feeling ‘better.’ Physical benefits included reduced symptoms such as breathlessness, pain and fatigue; improved symptom self-management, physical function, functional capacity, fitness, muscle strength and/or tone, and flexibility; and prevention of deterioration/functional maintenance. Mental benefits included improved self-esteem, self-efficacy, coping skills, relaxation, cognition, positivity, emotional regulation, and reduced stress and anxiety. | Moderate concerns  **Explanation:** Moderate concerns regarding methodological limitations because of concerns regarding researcher reflexivity across all included studies, data analysis reporting and/or rigour in 7 studies, data collection in 2 studies (including 1 that used clinician notes as a proxy for patient/caregiver experiences). This study did not contribute new knowledge to the finding, so the overall level of concern was rated as moderate. | No/Very minor concerns  **Explanation:** No/very minor concerns regarding coherence because the finding reflects the varied and complex data of the supporting studies. | No/Very minor concerns  **Explanation:** No/very minor concerns regarding adequacy because 17 studies contributed to this finding, including a rich body of supporting evidence. | No/Very minor concerns  **Explanation:** No/very minor concerns regarding relevance because while 2 studies did not specify the program provider, all other contextual factors were relevant to those specified in the review question. | High confidence  **Explanation:** Due to moderate concerns regarding methodological limitations and no/very minor concerns regarding coherence, adequacy, and relevance. | (1) (2) (3) (4) (6) (7) (11) (12) (13) (14) (15) (16) (17) (19) (20) (22) (23) |
| 22 | Many participants generated increased exercise self-efficacy through repeated exercise practice and supported exercise skill-building. | Moderate concerns  **Explanation:** Moderate concerns regarding methodological limitations because of concerns relating to researcher reflexivity across all supporting studies and data analysis reporting and/or rigour in 4 studies. | Moderate concerns  **Explanation:** Moderate concerns regarding coherence because the increase in self-efficacy could be explained by other factors such as goal attainment or encouragement from health professionals. | Minor concerns  **Explanation:** Minor concerns regarding adequacy because not all of the data provided detail regarding the reasons behind increased self-efficacy. Given that the finding is descriptive, the concern surrounding adequacy was considered minor. | No/Very minor concerns  **Explanation:** No/very minor concerns regarding relevance because while 2 studies did not specify the program provider, all other contextual factors were relevant to those specified in the review question. | Moderate confidence  **Explanation:** Due to moderate concerns regarding methodological limitations and coherence, minor concerns regarding adequacy, and no/very minor concerns regarding relevance. | (2) (4) (6) (7) (13) (15) (16) (17) (20) (22) |
| 23 | All stakeholder groups reported that caregivers benefitted broadly from participating and/or supporting their loved ones, including increased motivation to be physically active themselves, improved physical and/or mental well-being, and an opportunity to support and/or re-build relationships with their loved one. They also reported improved knowledge, such as an increased understanding of their loved ones’ health and diagnosis, and of ways to support and care for their loved ones. | Moderate concerns  **Explanation:** Moderate concerns regarding methodological limitations because of concerns relating to researcher reflexivity, data collection methods in 2 studies (including 1 study that used clinician documentation as a proxy for patient/caregiver experiences), and data analysis reporting and/or rigour in 1 study. | Moderate concerns  **Explanation:** Moderate concerns regarding coherence because only 3 supporting studies provided data specific to the caregiver's perspective. Because the majority of studies report from the patient's perspective (about the caregivers), this raises concerns due to the potential of oversimplification of descriptions. | Minor concerns  **Explanation:** Minor concerns regarding adequacy because whilst only 8 studies supported the finding, the data was moderately rich, and the finding is relatively descriptive. | Moderate concerns  **Explanation:** Moderate concerns regarding relevance because only 3 of the included studies reported formally including family members/caregivers in the exercise program, and only 2 reported formally collecting data from family members/caregivers themselves, with the others using patient and/or provider recounts as a proxy for their perspectives. | Moderate confidence  **Explanation:** Due to moderate concerns regarding methodological limitations, coherence and relevance, and minor concerns regarding adequacy. | (4) (12) (16) (18) (19) (20) (22) (23) |
| 24 | Some participants of pre- and rehabilitation programs believed that exercise was a significant driver in their surgical readiness and improving and/or hastening their postoperative recovery. | Minor concerns  **Explanation:** Minor concerns regarding methodological limitations because of concerns regarding researcher reflexivity, and recruitment transparency and data analysis reporting and/or rigour in 1 study. | No/Very minor concerns  **Explanation:** No/very minor concerns regarding coherence because the finding reflects the variation of perspectives discussed in the data. | Minor concerns  **Explanation:** Minor concerns regarding adequacy because whilst there are only 5 supporting studies, the data is adequately rich to support the descriptive finding. | No/Very minor concerns  **Explanation:** No/very minor concerns regarding relevance because the contextual factors were largely relevant to those specified in the review question. 1 study did not specify the program provider, and 1 contributing study focused on an inoperable lung cancer population. However, the patient whose perspective contributed to the finding was an outlier who progressed to having surgery. | High confidence  **Explanation:** Due to minor concerns regarding methodological limitations and adequacy and no/very minor concerns regarding coherence and relevance. | (2) (4) (6) (13) (14) |
| 25 | Most, but not all, participants felt that they benefited from participating in programs. Providers also believed that programs were beneficial. Some participants reported no perceived benefits, and others felt they would have been active despite their enrolment in a program and, therefore, that the program was unnecessary for them. | Serious concerns  **Explanation:** Serious concerns regarding methodological limitations because of concerns related to reflexivity across all studies, reporting and/or rigour of analysis in 7 studies, data collection in 1 study, and recruitment transparency in 3 studies. | No/Very minor concerns  **Explanation:** No/very minor concerns regarding coherence because the finding captures the variety and complexity of data. | No/Very minor concerns  **Explanation:** No/very minor concerns regarding adequacy because 12 studies contributed to this finding, and the supporting data was moderately rich and sufficient for the descriptive nature of the finding. | No/Very minor concerns  **Explanation:** No/very minor concerns regarding relevance because while 1 study did not specify the program provider, all other contextual factors were relevant to those specified in the review question. | Moderate confidence  **Explanation:** Due to serious concerns regarding methodological limitations and no/very minor concerns regarding coherence, adequacy, and relevance. | (1) (2) (3) (4) (5) (6) (7) (12) (14) (15) (17) (19) |
| Theme 4: Facilitating exercise behaviour change | | | | | | | |
| *Key enablers of exercise* | | | | | | | |
| 26 | Generating exercise habits and finding ways to integrate exercise into the daily routine were key enablers of lasting behaviour change. Program structure and a requirement to attend scheduled sessions were key extrinsic motivating factors for participation for some participants, whereas for others, the flexibility home-based programs offered to choose when to exercise was an enabler. | Moderate concerns  **Explanation:** Moderate concerns regarding methodological limitations because of concerns raised about researcher reflexivity across all supporting studies, data analysis reporting and/or rigour in 4 studies, and recruitment transparency in 1 study. | No/Very minor concerns  **Explanation:** No/very minor concerns regarding coherence because the review finding is descriptive in nature and reflects the varied and complex supporting data. | No/Very minor concerns  **Explanation:** No/very minor concerns regarding adequacy because 12 studies contributed data of varying richness adequate to support the descriptive finding. | No/Very minor concerns  **Explanation:** No/very minor concerns regarding relevance because while 2 studies did not specify the program provider, all other contextual factors were relevant to those specified in the review question. | High confidence  **Explanation:** Due to moderate concerns regarding methodological limitations and no/very minor concerns regarding coherence, adequacy, and relevance. | (1) (2) (4) (5) (6) (7) (11) (12) (13) (19) (20) (22) |
| 27 | Program providers were seen as key enablers of participation. Many participants felt accountable or committed to their providers, facilitating adherence. Participants of centre-based programs reported relying on external supervision to sustain motivation. Conversely, for participants of home-based programs, regular remote reviews with providers, such as phone calls, served a similar purpose. Across all exercise settings, participants valued receiving feedback on progress and activity levels from providers. Education and reassurance from expert providers regarding the importance and safety of exercise were critical enablers of exercise uptake and adherence. | Serious concerns  **Explanation:** Serious concerns regarding methodological limitations because of concerns relating to researcher reflexivity in all supporting studies, data analysis reporting and/or rigour in 7, recruitment transparency in 4 studies, and methodology appropriateness in 2. | No/Very minor concerns  **Explanation:** No/very minor concerns regarding coherence because the finding reflects the complexity and variation within the data. | No/Very minor concerns  **Explanation:** No/very minor concerns regarding adequacy because 15 studies contributed data of varying richness adequate to support the descriptive finding. | No/Very minor concerns  **Explanation:** No/very minor concerns regarding relevance because while 2 studies did not specify the program provider, all other contextual factors were relevant to those specified in the review question. | Moderate confidence  **Explanation:** Due to serious concerns regarding methodological limitations, and no/very minor concerns regarding coherence, adequacy, and relevance. | (1) (2) (4) (5) (6) (7) (8) (9) (12) (13) (15) (17) (19) (22) (24) |
| 28 | Clear activity goals (e.g., daily step targets) can drive behaviour change, and many participants saw activity monitoring tools such as pedometers as critical sources of extrinsic motivation. These tools provided a visual tool, enabled the self-monitoring of activity levels and recovery, and served as proof of ability. Achieving and/or exceeding exercise goals was an important driver of self-efficacy and a sense of achievement. | Serious concerns  **Explanation:** Serious concerns regarding methodological limitations because of concerns relating to researcher reflexivity across all studies, recruitment transparency in 3 studies, data collection in 2 studies, and data analysis reporting and/or rigour in 5 studies. | Minor concerns  **Explanation:** Minor concerns regarding coherence because Minor concerns regarding coherence because this finding does not discuss variation/outliers who did not enjoy activity monitors. This is discussed in a future finding. | No/Very minor concerns  **Explanation:** No/very minor concerns regarding adequacy because 14 studies supported the finding, with data varied from superficial to rich. Overall, the data provided sufficient detail to support the finding. | No/Very minor concerns  **Explanation:** No/very minor concerns regarding relevance because while 1 study did not specify the program provider, all other contextual factors were relevant to those specified in the review question. | Moderate confidence  **Explanation:** Due to serious concerns regarding methodological limitations, minor concerns regarding coherence, and no/very minor concerns regarding adequacy and relevance. | (4) (5) (6) (8) (9) (11) (12) (13) (14) (17) (19) (20) (22) (23) |
| 29 | Positive outcome expectancy was key to motivation and hope among many participants. Participants and providers tended to report a shared understanding of the importance and efficacy of exercise for patients with lung cancer and other medical conditions. Believing that the program would positively influence health, well-being or longevity, and/or prevent deterioration/decline, was a strong enabler of program sign-up and adherence, and experiencing perceived benefits of participation was additionally a strong enabler of adherence/sustained exercise uptake. | Serious concerns  **Explanation:** Serious concerns regarding methodological limitations because of concerns relating to the appropriateness of methodology in 2 studies, recruitment transparency in 3 studies, data collection appropriateness and reporting in 3 studies, researcher reflexivity in all studies, and data analysis reporting and/or rigour in 7 studies. | No/Very minor concerns  **Explanation:** No/very minor concerns regarding coherence because the finding reflects the variation in the data. | No/Very minor concerns  **Explanation:** No/very minor concerns regarding adequacy because 17 studies contributed to this finding with sufficient richness. | No/Very minor concerns  **Explanation:** No/very minor concerns regarding relevance because the contextual factors were relevant to those specified in the review question. | Moderate confidence  **Explanation:** Due to serious concerns regarding methodological limitations, minor concerns regarding coherence, and no/very minor concerns regarding adequacy and relevance. | (1) (2) (3) (4) (6) (7) (8) (12) (13) (14) (15) (16) (17) (19) (21) (23) (24) |
| 30 | Many participants saw having pre-existing exercise habits before their lung cancer diagnosis as a key enabler to recommencing and sustaining activity, exercise self-efficacy, and increased exercise intensity, particularly when facing barriers. | Minor concerns  **Explanation:** Minor concerns regarding methodological limitations because of concerns relating to data collection methods in 1 study, and data analysis rigor/reporting in 2 others. Reflexivity was not adequately discussed across all included studies, but researcher role was judged as unlikely to greatly influence this finding, so the overall concern was rated as minor. | Minor concerns  **Explanation:** Minor concerns regarding coherence because in one study, while pre-existing habits enabled exercise participation, this was reported as a tendency to 'overexert' oneself. | Minor concerns  **Explanation:** Minor concerns regarding adequacy because while only 7 studies supported the finding, supporting data was generally rich and provided sufficient detail for this descriptive finding. | No/Very minor concerns  **Explanation:** No/very minor concerns regarding relevance because the contextual factors were relevant to those specified in the review question. | High confidence  **Explanation:** Due to minor concerns regarding methodological limitations, coherence, and adequacy and no/very minor concerns regarding relevance. | (1) (4) (6) (13) (17) (19) (23) |
| 31 | Optimising symptom control and support to monitor and self-manage symptoms in collaboration with providers and other healthcare professionals enabled exercise uptake and adherence. Some participants displayed an inherent persistence and self-efficacy to exercise despite severe symptoms and side effects, while others generated this through the support of their providers. | Moderate concerns  **Explanation:** Moderate concerns regarding methodological limitations because 1 study had concerns relating to recruitment transparency, 1 relating to data collection appropriateness, 3 relating to data analysis reporting and/or rigour, and all had varying degrees of concern relating to reflexivity. | No/Very minor concerns  **Explanation:** No/very minor concerns regarding coherence because the finding reflects the complexity and variation of the data. | No/Very minor concerns  **Explanation:** No/very minor concerns regarding adequacy because 12 studies provided rich supporting data. | No/Very minor concerns  **Explanation:** No/very minor concerns regarding relevance because while 2 studies did not specify the program provider, all other contextual factors were relevant to those specified in the review question. | High confidence  **Explanation:** Due to moderate concerns regarding methodological limitations and no/very minor concerns regarding coherence, adequacy, and relevance. | (1) (2) (4) (6) (7) (10) (12) (13) (14) (19) (22) (23) |
| 32 | Encouragement and support from loved ones increased motivation for some participants. Participating in exercise alongside loved ones also increased motivation and enjoyment. | Minor concerns  **Explanation:** Minor concerns regarding methodological limitations because of concerns relating to researcher reflexivity across all studies, which was judged as unlikely to influence this finding greatly, concerns regarding data collection in 1 study, and data analysis in another. | No/Very minor concerns  **Explanation:** No/very minor concerns regarding coherence because the descriptive finding broadly reflects the supporting data. | Minor concerns  **Explanation:** Minor concerns regarding adequacy because only 8 studies contributed to this finding, 4 of which contributed relatively superficial data. Concern was judged as minor due to the descriptive nature of the finding. | No/Very minor concerns  **Explanation:** No/very minor concerns regarding relevance because while 1 study did not specify the program provider, all other contextual factors were relevant to those specified in the review question. | High confidence  **Explanation:** Due to minor concerns regarding methodological limitations and adequacy and no/very minor concerns regarding coherence and relevance. | (4) (6) (13) (19) (21) (20) (22) (23) |
| *Key barriers to exercise* | | | | | | | |
| 33 | Symptoms such as fatigue, pain, and breathlessness were commonly experienced barriers to exercising. Adjuvant therapy side effects similarly often prevented participants from exercising, and participants recalled the ‘destructive’ nature of adjuvant therapies (especially chemotherapy) to both their bodies and their daily routines. Side effects associated with exercising such as exacerbating symptoms or inducing muscle pain/fatigue, and comorbidities, also influenced participation. The impacts of these barriers varied, sometimes forcing exercise regression or modification and/or completely inhibiting participation. | Serious concerns  **Explanation:** Serious concerns regarding methodological limitations because of concerns relating to recruitment transparency in 2 studies, data collection in 1 study, data analysis in 5 studies, and reflexivity across all studies. | No/Very minor concerns  **Explanation:** No/very minor concerns regarding coherence because the finding reflects the variation and complexity of the data. | No/Very minor concerns  **Explanation:** No/very minor concerns regarding adequacy because 12 studies supported this finding, 4 of which provided thin supporting data. The remaining 8 studies are rich and provide sufficient detail to understand the phenomenon of interest. | No/Very minor concerns  **Explanation:** No/very minor concerns regarding relevance because while 1 study did not specify the program provider, all other contextual factors were relevant to those specified in the review question. | Moderate confidence  **Explanation:** Due to serious concerns regarding methodological limitations and no/very minor concerns regarding coherence, adequacy, and relevance. | (1) (6) (8) (11) (13) (14) (15) (17) (19) (20) (21) (23) |
| 34 | Time was a barrier to exercising. The unpredictability of cancer treatment pathways, particularly planned and unplanned medical appointments, influenced participants’ ability to participate in both home and centre-based programs. Additionally, exercise was often deprioritised in favour of other competing priorities, such as employment, caregiving, and socialising. | Moderate concerns  **Explanation:** Moderate concerns regarding methodological limitations because of concerns relating to data collection in 1 study, data analysis reporting and/or rigour in 3 studies, and researcher reflexivity in all studies. | No/Very minor concerns  **Explanation:** No/very minor concerns regarding coherence because the finding is descriptive and captures the breadth and variety of data represented in the supporting studies. | Minor concerns  **Explanation:** Minor concerns regarding adequacy because 4/8 included studies provide relatively superficial data. Considering the descriptive nature of the finding, the level of concern was judged to be minor. | No/Very minor concerns  **Explanation:** No/very minor concerns regarding relevance because while 1 study did not specify the program provider, all other contextual factors were relevant to those specified in the review question. | High confidence  **Explanation:** Due to moderate concerns regarding methodological limitations, no/very minor concerns regarding coherence and relevance, and minor concerns regarding adequacy. | (4) (6) (7) (8) (17) (19) (20) (23) |
| 35 | Poor weather (e.g., rain, wind and extreme heat and/or cold) inhibited many participants' ability and/or willingness to participate in home-based exercise. | Minor concerns  **Explanation:** Minor concerns regarding methodological limitations because of concerns regarding recruitment transparency in 1 study, data collection in 1 study, and data analysis in 1 study. Reflexivity was a concern across all studies, but was not judged to likely influence this finding, and so the overall concern was rated as minor. | No/Very minor concerns  **Explanation:** No/very minor concerns regarding coherence because the finding reflects the varied data of the underlying studies. | Moderate concerns  **Explanation:** Moderate concerns regarding adequacy because only 5 studies, with relatively thin data, contributed to this descriptive finding. | No/Very minor concerns  **Explanation:** No/very minor concerns regarding relevance because the contextual factors were relevant to those specified in the review question. | High confidence  **Explanation:** Due to moderate concerns regarding methodological limitations and adequacy and no/very minor concerns regarding coherence and relevance. | (6) (8) (13) (15) (23) |
| 36 | For participants of one study, social and cultural beliefs about emphasising rest in recovery were a barrier to postoperative exercise. | No/Very minor concerns  **Explanation:** No/very minor concerns regarding methodological limitations because of concerns relating to researcher reflexivity, although researcher role was not judged as likely influence this finding, and so the overall concern was rated as very minor. | Moderate concerns  **Explanation:** Moderate concerns regarding coherence because the data supporting the finding were not varied and represent the dominant theme identified in one study. | Serious concerns  **Explanation:** Serious concerns regarding adequacy because only one study contributed to this finding. The supporting data of the one contributing study is moderately rich and provides details to understand the phenomenon. However, when considering the quantity of data, the level of concern is raised to serious. | No/Very minor concerns  **Explanation:** No/very minor concerns regarding relevance because while 1 study did not specify the program provider, all other contextual factors were relevant to those specified in the review question. | Low confidence  **Explanation:** Due to no/very minor concerns regarding methodological limitations and relevance, moderate concerns regarding coherence, and serious concerns regarding adequacy. | (13) |
| 37 | Participants recalled difficulty generating the intrinsic motivation required to ‘get moving’, often citing their own ‘laziness’ or lack of discipline as a barrier. Low exercise self-efficacy similarly prevented some participants from exercising. Participants of centre-based programs especially often reported challenges initiating independent exercise outside of their supervised sessions due to lacking motivation and/or confidence. | Serious concerns  **Explanation:** Serious concerns regarding methodological limitations because of concerns relating to methodological appropriateness in 1 study, recruitment transparency in 1 study, data collection methods in 2 studies, data analysis reporting and/or rigour in 5 studies, and researcher reflexivity across all studies. | No/Very minor concerns  **Explanation:** No/very minor concerns regarding coherence because the finding is descriptive in nature and reflects the varied intrinsic barriers to exercise participation. | Minor concerns  **Explanation:** Minor concerns regarding adequacy because while 13 studies contributed, data was sometimes thin. Given the descriptive nature of the finding, the level of concern was judged to be minor. | No/Very minor concerns  **Explanation:** No/very minor concerns regarding relevance because the contextual factors were relevant to those specified in the review question. | Moderate confidence  **Explanation:** Due to moderate concerns regarding methodological limitations, no/very minor concerns regarding coherence and relevance, and minor concerns regarding adequacy. | (1) (2) (4) (6) (7) (13) (15) (16) (17) (19) (20) (22) (23) |
| 38 | Pre-existing habits relating to exercise influenced participation. Participants who reported having a relatively sedentary lifestyle before commencing their program felt this was a barrier to exercise uptake. In contrast, some participants who viewed themselves as active felt that an exercise program would not necessarily benefit them. | Minor concerns  **Explanation:** Minor concerns regarding methodological limitations because of concerns relating to reflexivity in all studies, data collection in 1 study, and data analysis in another. | Minor concerns  **Explanation:** Minor concerns regarding coherence because outliers are described alongside the dominant pattern found in the data. | Moderate concerns  **Explanation:** Moderate concerns regarding adequacy because only 4 studies contributed to this finding, with insufficiently rich data to support the entire finding in adequate depth. | No/Very minor concerns  **Explanation:** No/very minor concerns regarding relevance because while 1 study did not specify the program provider, all other contextual factors were relevant to those specified in the review question. | Moderate confidence  **Explanation:** Due to minor concerns regarding methodological limitations and coherence, moderate concerns regarding adequacy, and no/very minor concerns regarding relevance. | (1) (2) (4) (23) |
| 39 | Program acceptability was a barrier to exercise participation, including participants’ lack of enjoyment of exercise, preference to remain sedentary, and/or disinterest in the type of exercise prescribed. A minority of participants felt that they did not require such a program, and/or that it would not help them. Some participants and providers also questioned program appropriateness at times, for example, the appropriateness of prescribing high-intensity exercise to participants early after surgery or those with short life expectancies. | Moderate concerns  **Explanation:** Moderate concerns regarding methodological limitations because of concerns relating to recruitment transparency in 1 study, data collection in 1 study, data analysis reporting and/or rigor in 3 studies, and reflexivity across all studies. | No/Very minor concerns  **Explanation:** No/very minor concerns regarding coherence because the finding reflects the complexity and variation of the data. | Minor concerns  **Explanation:** Minor concerns regarding adequacy because the supporting data was superficial in 2 studies. Considering the finding is descriptive in nature, the level of concern was judged to be minor. | No/Very minor concerns  **Explanation:** No/very minor concerns regarding relevance because while 1 study did not specify the program provider, all other contextual factors were relevant to those specified in the review question. | High confidence  **Explanation:** Due to moderate concerns regarding methodological limitations, no/very minor concerns regarding coherence and relevance, and minor concerns regarding adequacy. | (1) (6) (8) (12) (15) (19) (20) (23) |
| 40 | Participants and providers encountered several logistical barriers, often specific to program design. Participants of centre-based programs were limited by travel and parking requirements, with some living considerable distances away. Prehabilitation programs were limited by the limited time between diagnosis and surgery. | Moderate concerns  **Explanation:** Moderate concerns regarding methodological limitations because of concerns relating to data analysis reporting and/or rigor in 2 studies, recruitment transparency in 1 study, and reflexivity across all studies. | Minor concerns  **Explanation:** Minor concerns regarding coherence because the finding does not wholly reflect the complexity around transport requirements (e.g., mobility concerns and non-car transport). | Moderate concerns  **Explanation:** Moderate concerns regarding adequacy because only 5 studies contributed, with 2 providing very superficial data. | No/Very minor concerns  **Explanation:** No/very minor concerns regarding relevance because while 1 study did not specify the program provider, all other contextual factors were relevant to those specified in the review question. | Moderate confidence  **Explanation:** Due to moderate concerns regarding methodological limitations and adequacy, minor concerns regarding coherence, and no/very minor concerns regarding relevance. | (2) (4) (6) (15) (20) |
| 41 | Participants of programs incorporating a technological element, such as online symptom monitoring or activity tracking technologies, encountered specific barriers, such as a lack of digital skills and/or self-efficacy, discomfort, and a general dislike of the prescribed technology. | Minor concerns  **Explanation:** Minor concerns regarding methodological limitations because of concerns in 1 study regarding recruitment transparency and data analysis reporting and/or rigor, and regarding reflexivity across all studies. | No/Very minor concerns  **Explanation:** No/very minor concerns regarding coherence because the finding reflects the variation in the data. | Minor concerns  **Explanation:** Minor concerns regarding adequacy because 7 studies contributed to the finding, with 3 providing thin data. Considering the descriptive nature of the finding and the presence of rich data across other studies, the level of concern was rated to be minor. | No/Very minor concerns  **Explanation:** No/very minor concerns regarding relevance because the contextual factors were relevant to those specified in the review question. | High confidence  **Explanation:** Due to minor concerns regarding methodological limitations and adequacy, and no/very minor concerns regarding coherence and relevance. | (6) (8) (12) (13) (15) (19) (22) |

**References**

1. Harris JL, Booth A, Cargo M, Hannes K, Harden A, Flemming K, Garside R, Pantoja T, Thomas J, Noyes J (2018) Cochrane Qualitative and Implementation Methods Group guidance series—paper 2: methods for question formulation, searching, and protocol development for qualitative evidence synthesis J Clin Epidemiol 97: 39-48. <https://doi.org/10.1016/j.jclinepi.2017.10.023>

2. Soilemezi D, Linceviciute S (2018) Synthesizing Qualitative Research: Reflections and Lessons Learnt by Two New Reviewers International Journal of Qualitative Methods 17 (1): 1609406918768014. <https://doi.org/10.1177/1609406918768014>

3. Jordan Z, Lockwood C, Munn Z, Aromataris E (2019) The updated Joanna Briggs Institute Model of Evidence-Based Healthcare JBI Evidence Implementation 17 (1)

4. Higgins JPT, Thomas J, Chandler J, Cumpston M, Li T PM, Welch VA (2021) Cochrane Handbook for Systematic Reviews of Interventions Version 6.2 (updated February 2021). Cochrane

5. The InterTASC Information Specialists' Sub-Group Qualitative Research: Filters. <https://sites.google.com/a/york.ac.uk/issg-search-filters-resource/home/qualitative-research>. 8th of October 2024

6. Bramer WM, Rethlefsen ML, Kleijnen J, Franco OH (2017) Optimal database combinations for literature searches in systematic reviews: a prospective exploratory study Systematic Reviews 6 (1): 245. <https://doi.org/10.1186/s13643-017-0644-y>

7. Haddaway NR, Collins AM, Coughlin D, Kirk S (2015) The Role of Google Scholar in Evidence Reviews and Its Applicability to Grey Literature Searching PLoS One 10 (9): e0138237. 10.1371/journal.pone.0138237

8. Booth A, Harris J, Croot E, Springett J, Campbell F, Wilkins E (2013) Towards a methodology for cluster searching to provide conceptual and contextual “richness” for systematic reviews of complex interventions: case study (CLUSTER) BMC Med Res Methodol 13 (1): 118. 10.1186/1471-2288-13-118

9. Noyes J, Booth A, Lewin S, Carlsen B, Glenton C, Colvin CJ, Garside R, Bohren MA, Rashidian A, Wainwright M, Tunςalp Ö, Chandler J, Flottorp S, Pantoja T, Tucker JD, Munthe-Kaas H (2018) Applying GRADE-CERQual to qualitative evidence synthesis findings–paper 6: how to assess relevance of the data Implement Sci 13 (1): 4. 10.1186/s13012-017-0693-6

10. Lewin S, Bohren M, Rashidian A, Munthe-Kaas H, Glenton C, Colvin CJ, Garside R, Noyes J, Booth A, Tunçalp Ö, Wainwright M, Flottorp S, Tucker JD, Carlsen B (2018) Applying GRADE-CERQual to qualitative evidence synthesis findings—paper 2: how to make an overall CERQual assessment of confidence and create a Summary of Qualitative Findings table Implement Sci 13 (1): 10. 10.1186/s13012-017-0689-2

11. Munthe-Kaas H, Bohren MA, Glenton C, Lewin S, Noyes J, Tunçalp Ö, Booth A, Garside R, Colvin CJ, Wainwright M, Rashidian A, Flottorp S, Carlsen B (2018) Applying GRADE-CERQual to qualitative evidence synthesis findings—paper 3: how to assess methodological limitations Implement Sci 13 (1): 9. 10.1186/s13012-017-0690-9

12. Colvin CJ, Garside R, Wainwright M, Munthe-Kaas H, Glenton C, Bohren MA, Carlsen B, Tunçalp Ö, Noyes J, Booth A, Rashidian A, Flottorp S, Lewin S (2018) Applying GRADE-CERQual to qualitative evidence synthesis findings—paper 4: how to assess coherence Implement Sci 13 (1): 13. 10.1186/s13012-017-0691-8

13. Glenton C, Carlsen B, Lewin S, Munthe-Kaas H, Colvin CJ, Tunçalp Ö, Bohren MA, Noyes J, Booth A, Garside R, Rashidian A, Flottorp S, Wainwright M (2018) Applying GRADE-CERQual to qualitative evidence synthesis findings—paper 5: how to assess adequacy of data Implement Sci 13 (1): 14. 10.1186/s13012-017-0692-7

14. Lewin S, Booth A, Glenton C, Munthe-Kaas H, Rashidian A, Wainwright M, Bohren MA, Tunçalp Ö, Colvin CJ, Garside R, Carlsen B, Langlois EV, Noyes J (2018) Applying GRADE-CERQual to qualitative evidence synthesis findings: introduction to the series Implementation Science 13 (1): 2. <https://doi.org/10.1186/s13012-017-0688-3>

15. Norwegian Institute of Public Health (developed by the Epistemonikos Foundation MWCatNIoPHftG-CPG (2025) GRADE-CERQual Interactive Summary of Qualitative Findings (iSoQ).isoq.epistemonikos.org

16. Wainwright M, Zahroh RI, Tunçalp Ö, Booth A, Bohren MA, Noyes J, Cheng W, Munthe-Kaas H, Lewin S (2023) The use of GRADE-CERQual in qualitative evidence synthesis: an evaluation of fidelity and reporting Health Res Policy Syst 21 (1): 77. 10.1186/s12961-023-00999-3

17. Page MJ, McKenzie JE, Bossuyt PM, Boutron I, Hoffmann TC, Mulrow CD, Shamseer L, Tetzlaff JM, Akl EA, Brennan SE, Chou R, Glanville J, Grimshaw JM, Hróbjartsson A, Lalu MM, Li T, Loder EW, Mayo-Wilson E, McDonald S, McGuinness LA, Stewart LA, Thomas J, Tricco AC, Welch VA, Whiting P, Moher D (2021) The PRISMA 2020 statement: an updated guideline for reporting systematic reviews BMJ 372: n71. <https://doi.org/10.1136/bmj.n71>

18. Tong A, Flemming K, McInnes E, Oliver S, Craig J (2012) Enhancing transparency in reporting the synthesis of qualitative research: ENTREQ BMC Med Res Methodol 12 (1): 181. <https://doi.org/10.1186/1471-2288-12-181>

19. UTHealth Houston School of Public Health (2021) Search Filters for Various Databases. <https://libguides.sph.uth.tmc.edu/c.php?g=543319&p=3723017>. 8th of October 2024

20. Wagner M, Rosumeck S, Küffmeier C, Döring K, Euler U (2020) A validation study revealed differences in design and performance of MEDLINE search filters for qualitative research J Clin Epidemiol 120: 17-24. 10.1016/j.jclinepi.2019.12.008

21. Hoffmann TC, Glasziou PP, Boutron I, Milne R, Perera R, Moher D, Altman DG, Barbour V, Macdonald H, Johnston M, Lamb SE, Dixon-Woods M, McCulloch P, Wyatt JC, Chan A-W, Michie S (2014) Better reporting of interventions: template for intervention description and replication (TIDieR) checklist and guide BMJ 348
